# Impaired nucleocytoplasmic transport in SOD1-mediated ALS

**DOI:** 10.1186/s13024-026-00930-8

**Published:** 2026-02-14

**Authors:** Shirel Argueti-Ostrovsky, Su Min Lim, Olubankole A. Arogundade, Sandra Diaz-Garcia, Gulshan Yunisova, Alex Meng, Anita Hermann, Kailee Ong, Ekaterina Eremenko, Mariana Bravo-Hernandez, Shawn P. Driscoll, Chao-Zong Lee, Xin Jiang, Alexandra Stavsky, Shir Barel, Tom Shani, Joy Kahn, Samuel L. Pfaff, Alon Monsonego, Martin Marsala, John Ravits, Clotilde Lagier-Tourenne, Adrian Israelson

**Affiliations:** 1 Department of Physiology and Cell Biology, Faculty of Health Sciences, Ben-Gurion University of the Negev, P.O.B. 653 84105, Beer Sheva, Israel; 2 The School of Brain Sciences and Cognition, Ben-Gurion University of the Negev, P.O.B. 653, Beer Sheva, 84105 Israel; 3 Department of Neurology, the Sean M. Healey and AMG Center for ALS at Mass General, Massachusetts General Brigham, Harvard Medical School, Boston, MA 02114 USA; 4 Broad Institute of Harvard University and MIT, Cambridge, MA 02142 USA; 5 Department of Neurosciences, University of California, San Diego, La Jolla, CA 92093 USA; 6 Department of Anesthesiology, School of Medicine, University of California San Diego, San Diego, CA USA; 7 Gene Expression Laboratory and the Howard Hughes Medical Institute, Salk Institute for Biological Studies, La Jolla, CA USA; 8 The Shraga Segal Department of Microbiology, Immunology and Genetics, Faculty of Health Sciences, Ben-Gurion University of the Negev, Beer Sheva, Israel; 9 The Regenerative Medicine and Stem Cell Research Center, Ben-Gurion University of the Negev, Beer Sheva, Israel; 10 National Institute for Biotechnology in the Negev, Ben-Gurion University of the Negev, Beer Sheva, Israel

**Keywords:** Nucleocytoplasmic transport, Nuclear pore complex, ALS, Misfolded SOD1, Neurodegeneration, RanGAP1, XPO1, Nucleoporins

## Abstract

**Background:**

Impaired nucleocytoplasmic transport (NCT) has emerged as a shared pathogenic mechanism in various neurodegenerative disorders, including amyotrophic lateral sclerosis (ALS). Although mutations in the gene encoding superoxide dismutase 1 (SOD1) account for approximately 20% of familial ALS cases, the impact of mutant SOD1 accumulation on the NCT remains unclear.

**Methods:**

Utilizing in vitro and in vivo models, patient-derived fibroblasts, and postmortem spinal cord tissues from ALS patients with SOD1 mutations, we determined the effects of mutant SOD1 on NCT dynamics, nuclear morphology and cellular localization of transport receptors and nuclear pore components.

**Results:**

Mutant SOD1 disrupts nuclear import and export trafficking, causing cytosolic accumulation of key transport regulators such as RanGAP1 and exportin 1 (XPO1). Mutant SOD1 also lowers the abundance of FG-Nups at the nuclear pore without altering nuclear circularity. Abnormal accumulation of NCT components was identified in Iba1-positive microglia, indicating a previously overlooked, non-cell-autonomous contribution to disease pathogenesis. Importantly, AAV-mediated reduction of mutant SOD1 in transgenic mice restored nuclear XPO1 localization, underscoring the causal role of mutant SOD1 in NCT abnormalities. Finally, comparable NCT perturbations were observed in patient-derived fibroblasts and in post-mortem spinal cord tissues from individuals with SOD1-ALS.

**Conclusions:**

Our results implicate NCT disruption as a shared disease mechanism between SOD1-mediated ALS and other familial and sporadic forms of ALS, adding support for targeting this pathway as an attractive therapeutic strategy in this fatal disease.

**Supplementary information:**

The online version contains supplementary material available at 10.1186/s13024-026-00930-8.

## Background

Amyotrophic lateral sclerosis (ALS) is a fatal neurodegenerative disease characterized by the progressive degeneration of motor neurons in the brain and spinal cord, leading to muscle atrophy and paralysis, culminating in respiratory failure. Disease onset predominantly occurs around 40–60 years of age, with death typically following 3–5 years after diagnosis. However, some forms of the disease demonstrate prolonged survival. Approximately 90% of all ALS cases are sporadic (SALS), lacking an overt family history, while the remaining 10% are inherited usually as an autosomal dominant trait (familial ALS; FALS) [1]. The year 1993 marked the beginning of the molecular era in ALS research, with the identification of genetic mutations in the gene encoding superoxide dismutase 1 (SOD1) [2]. SOD1 is a cytosolic homodimeric metalloenzyme that catalyzes the dismutation of the toxic superoxide to oxygen and hydrogen peroxide [3]. SOD1 mutations are observed in ~20% of FALS cases [4] and ~2% of SALS cases [5]. Although initially assumed, the degeneration caused by mutant SOD1 is independent of dismutase activity and driven by the gain of one or more toxic properties as the mutant protein tends to misfold and form cytosolic aggregates [6–10].

Neurodegenerative diseases, and in particular ALS, are associated with protein misfolding and cytosolic accumulation of aggregation-prone proteins [11–13]. Cytosolic protein inclusions were shown to disrupt one of the most critical regulatory systems of the eukaryotic cell – the nucleocytoplasmic transport (NCT) machinery [14]. NCT refers to the selective passage of materials in and out of the nucleus via the nuclear pore complex (NPC). The mammalian NPC is a multi-protein structure comprising numerous copies of ~30 proteins called nucleoporins (Nups), organized into five main structural domains [15]. Several Nups provide structural support anchoring the NPC to the nuclear envelope, while approximately one-third contain phenylalanine-glycine repeat domains (also called FG-Nups), enabling the selective permeability barrier that regulates the trafficking of macromolecules through the pore. These low-complexity domains of the FG-Nups transiently bind to and interact with nuclear transport receptors (karyopherins), facilitating active passage of cargos. In general, molecules smaller than ~40 KDa can passively diffuse through the NPC. However, the transport of larger molecules relies on active transport facilitated by binding of a cargo protein to specific nuclear transport receptors via the recognition of nuclear localization and nuclear export sequences (NLS/NES, respectively) [16]. In the last decade, disruption of the NCT has been reported in several neurodegenerative diseases [17, 18] including ALS [18–33], frontotemporal dementia (FTD) [20, 22], Huntington’s disease [14, 34, 35], Alzheimer’s disease [36, 37], juvenile neurodegenerative disorders [15] and during aging [38, 39].

Several ALS-associated mutations were shown to compromise the NPC and the intact shuttling of material in and out of the nucleus. In particular, studies using Drosophila models of chromosome 9 open reading frame 72 (C9orf72), the most common genetic cause of ALS and frontotemporal dementia [40, 41] revealed that repeat-containing RNAs transcribed from the pathogenic C9orf72 G_4_C_2_ expansion sequesters RANGAP1, thereby disrupting the RAN-GTPase gradient, two critical components of the import process [19–21]. In addition, arginine (R)-rich dipeptide repeat proteins (DPR) translated from the G_4_C_2_ expansion alter nuclear import mediated by importin-β, the importin $\alpha$/$\beta$ complex and transportins (class of importins that recognizes the structurally distinct PY-NLS) [42]. All are critical nuclear transport receptors that promote nuclear import of many cargo-proteins including TAR DNA-binding protein 43 (TDP-43) [43] and fused in sarcoma (FUS) [44, 45]. Moreover, mutations in TDP-43, FUS and profilin 1 (PFN1), rare inherited forms of ALS, were associated with cytoplasmic mislocalization of Nups and NPC-associated proteins [22, 23, 27]. Here, we used both cellular and murine models to demonstrate that accumulation of cytosolic misfolded SOD1 is associated with impairment of the NCT. Mislocalization and nuclear depletion of nuclear transport receptors, as well as reduced levels of FG-Nups, were identified in mutant SOD1 mice, human-derived fibroblasts and spinal cord tissues from SOD1-ALS patients. We also provide the first evidence of aberrant accumulation of the nuclear transport machinery in non-neuronal cells, pointing to a previously overlooked non-cell-autonomous contributor to disease progression. Importantly, AAV-mediated silencing of mutant SOD1 was shown to alleviate cellular mislocalization of the export receptor XPO1 in mouse spinal motor neurons, supporting the notion that accumulated misfolded SOD1 impacts the integrity of NCT. Overall, our results identify disruption of NCT in SOD1-ALS and reinforce abnormal transport as a shared fundamental mechanism in ALS pathology.

## Results

### Mutant SOD1 impairs nuclear export

**Fig. 1:**
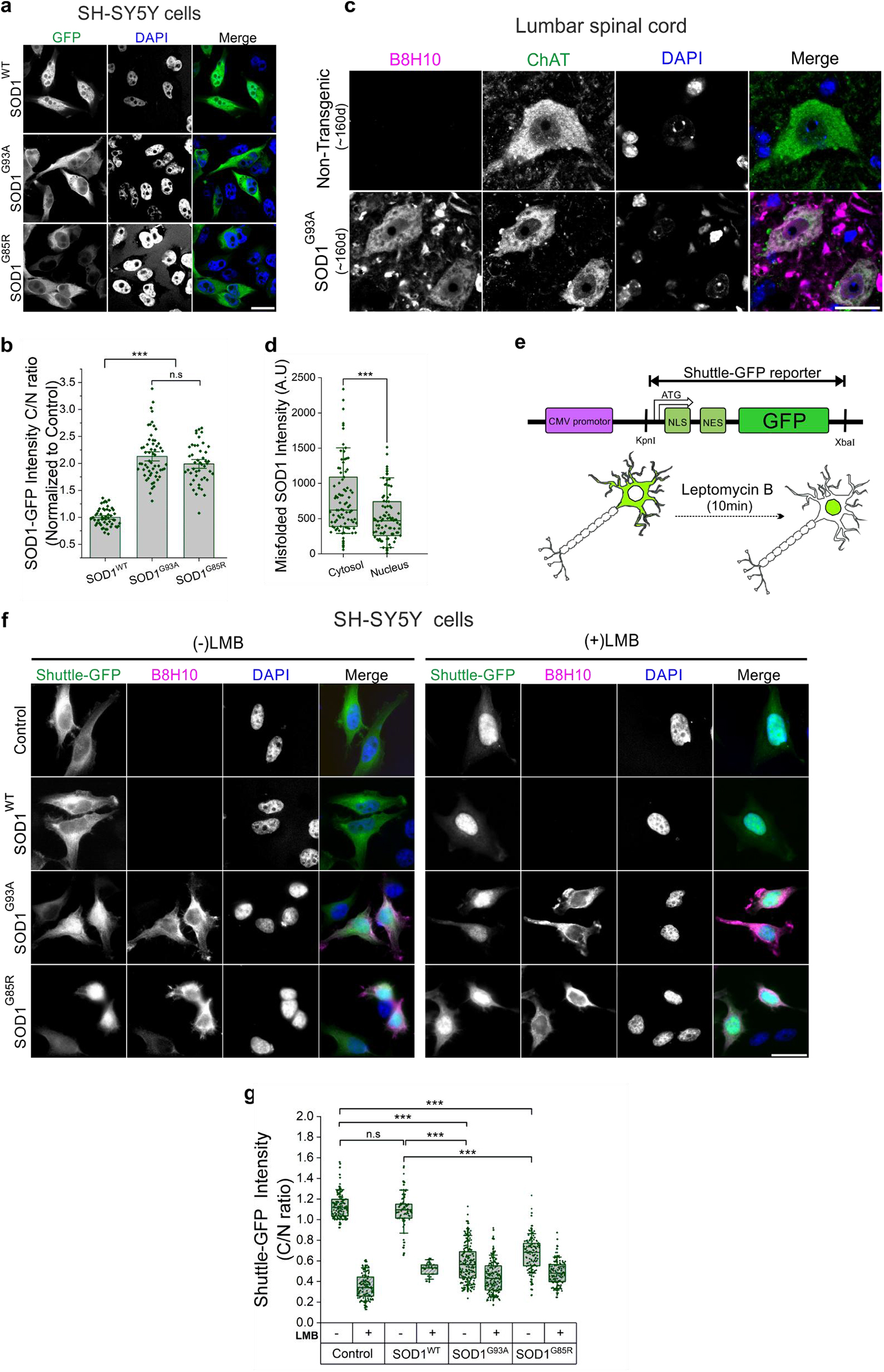
Cytosolic accumulation of misfolded SOD1 alters nucleocytoplasmic transport dynamics. **a**. Confocal imaging of SH-SY5Y cells expressing GFP-tagged SOD1^WT^, SOD1^G93A,^ or SOD1^G85R^ (green), and DAPI (blue) staining for nuclear identification. Scale bar, 20 µm. **b**. Quantification of cytosol-to-nucleus ratio of SOD1-GFP. **c**. Immunofluorescence of lumbar spinal cord sections from non-transgenic (*n* = 4) and end-stage SOD1^G93A^ (*n* = 4) mice stained with antibodies against B8H10 for misfolded SOD1 (magenta), ChAT (green), and DAPI (blue). Scale bar, 20 µm. **d**. Quantification of misfolded SOD1 in the cytosol and nucleus of spinal motor neurons from end-stage SOD1^G93A^ mice. **e**. Schematic domain structure of NLS-NES-GFP (Shuttle-GFP) construct and a diagram of NCT dynamics using exportin 1 inhibitor, leptomycin B (LMB), for 10 min. **f**. SH-SY5Y cells expressing Shuttle-GFP (green) and pCineo-empty plasmid (control), SOD1^WT^, SOD1^G93A^ or SOD1^G85R^. Accumulation of misfolded SOD1 was detected by B8H10 antibody (magenta), and nuclear boundaries were defined using DAPI (blue). 10 ng/ml LMB was added for 10 min where indicated. Scale bar, 20 µm. **g**. Quantification of Shuttle-GFP distribution in SH-SY5Y cells from data in (**f**). Bars represent mean ± SEM. Three independent experiments for (**b**) (dots represent cells from all experiments. Rank-based one-way ANOVA, p-values adjusted for multiple comparisons and clustering; n.S. non-significant, ***p < 0.001). Graphs represent quartiles (box), 50th percentiles (center lines) and range (10–90; whiskers). Four independent experiments for (**d**) (dots represent motor neurons from all experiments. Rank-based two-samples t-test, p-values adjusted for clustering; ***p < 0.001), three independent experiments for (**g**) (dots represent data from all the experiments. Rank-based one-way ANOVA, p-values adjusted for multiple comparisons and clustering. n.s. non-significant, ***p < 0.001)

SOD1 protein is normally localized in both the cytoplasm and the nucleus; however, ALS-associated mutant SOD1 is primarily cytosolic [46–51]. Indeed, Zhong and colleagues reported that misfolding of mutant SOD1 exposes a nuclear export signal (NES)-like sequence that is normally buried, leading to the accumulation of misfolded SOD1 in the cytoplasm [52]. Consistently, SOD1^WT^–EGFP expressed in SH-SY5Y cells was dispersed in both cellular compartments while the two mutants SOD1^G93A^–EGFP and SOD1^G85R^–EGFP localized predominantly in the cytoplasm (Fig. 1a, b). Similar cytoplasmic accumulation of misfolded SOD1 was observed when using a conformation-specific antibody (B8H10) in SH-SY5Y cells expressing untagged mutant SOD1 (Supplementary Fig. 1a, b) and in ChAT-positive motor neurons within the lumbar spinal cord of end-stage SOD1^G93A^ mice (Fig. 1c, d and Supplementary Fig. 1c). Additionally, another monoclonal antibody, SE-21, targeting the β6/β7 loop region, which is exposed in misfolded SOD1 [53, 54], revealed a similar staining pattern in ChAT-positive neurons from the lumbar spinal cord of symptomatic SOD1^G93A^ mice (Supplementary Fig. 1d, e).

It was proposed that protein aggregation in the cytoplasm, but not the nucleus, causes pronounced impairment of NCT and redistribution of nuclear-shuttling factors to the cytosol [14]. To determine whether cytoplasmic accumulation of misfolded SOD1 interferes with nuclear transport, we used an NLS-NES-GFP reporter (shuttle-GFP or S-GFP). Normally, S-GFP is mainly localized to the cytoplasm due to the dominant influence of the nuclear export signal (NES) compared to the nuclear localization signal (NLS) (Fig. 1e). Co-transfection of S-GFP and SOD1 mutants (SOD1^G93A^ or SOD1^G85R^) in SH-SY5Y cells led to abnormal distribution of the S-GFP protein with significant retention in the nucleus (Fig. 1f, g). In contrast, S-GFP remained predominantly accumulated in the cytosol of cells overexpressing SOD1^WT^, indicating inhibition of nuclear export in the presence of mutant misfolded SOD1. Moreover, the inhibition of the nuclear export receptor, exportin 1 (XPO1), by *leptomycin B* (LMB) caused S-GFP to accumulate in the nucleus of non-transfected or SOD1^WT^ expressing cells, displaying a similar phenotype to that observed in mutant SOD1 expressing cells prior to LMB induction (Fig. 1f, g). Treatment with LMB did not intensify the nuclear retention in mutant SOD1-expressing cells already manifesting a nuclear export defect. In conclusion, cytoplasmic accumulation of misfolded SOD1 interferes with the export of proteins through the nuclear pore.

### Altered XPO1 distribution correlates with misfolded SOD1 accumulation

**Fig. 2:**
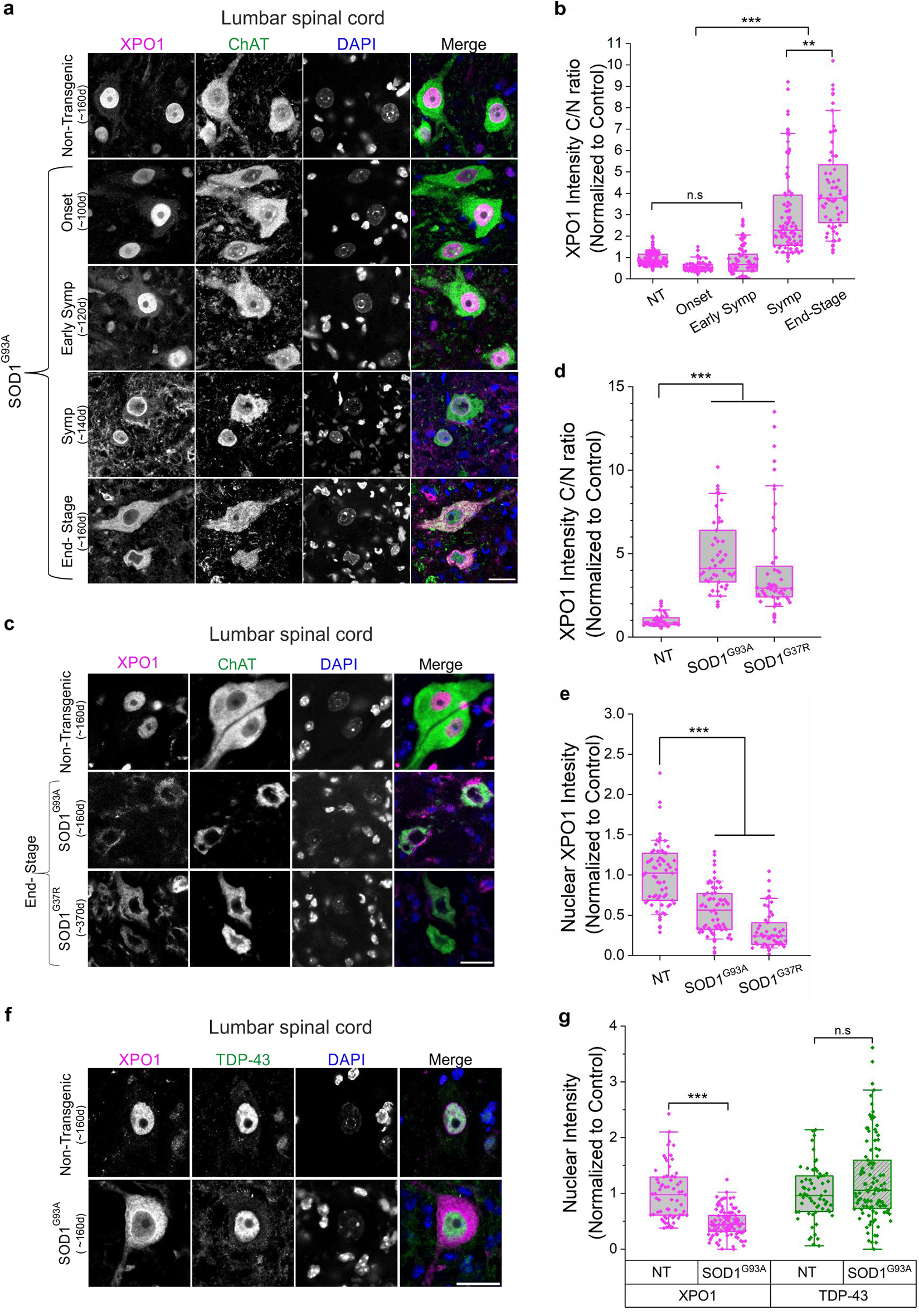
Cytoplasmic mislocalization and reduced nuclear levels of exportin 1 in the spinal cord of mutant SOD1 mice. **a**. Immunofluorescence of lumbar spinal cord sections from non-transgenic (*n* = 3) and mutant SOD1^G93A^ (*n* = 3) mice at different stages of the disease, stained with anti-XPO1 (magenta), anti-ChAT (green), and DAPI (blue). Scale bar, 20 µm. **b**. Quantification of cytosol-to-nucleus ratio of XPO1 distribution in spinal motor neurons from data in (**a**). **c**. Immunofluorescence of lumbar spinal cord sections from non-transgenic (*n* = 3), end-stage SOD1^G93A^ (*n* = 3) and SOD1^G37R^ (*n* = 4) mice stained with anti-XPO1 (magenta), anti-ChAT (green), and DAPI (blue). Scale bar, 20 µm. **d**. Quantification of cytosol-to-nucleus ratio of XPO1 distribution in spinal motor neurons from data in (**c**). **e**. Quantification of XPO1 nuclear levels in spinal motor neurons from data in (**c**). **f**. Immunofluorescence of XPO1 (magenta), TDP-43 (green) and DAPI (blue) in the ventral horn of lumbar spinal cord sections from non-transgenic (*n* = 3) and end-stage SOD1^G93A^ (*n* = 4) mice. Scale bar, 20 µm. **g**. Quantification of nuclear levels of XPO1 and TDP-43 represented in (**f**). Graphs represent quartiles (boxes) with data overlap, 50th percentiles (center lines) and range (10–90; whiskers). Three independent experiments for (**b**), (**d**), and (**e**) (dots represent quantified motor neurons from all experiments. Rank-based one-way ANOVA, p-values adjusted for multiple comparisons and clustering; n.s. non-significant, **p < 0.01, ***p < 0.001). Three independent experiments for (**g**) (dots represent quantified cells from all experiments. Rank-based one-way ANOVA, p-values adjusted for multiple comparisons and clustering; n.s. non-significant, ***p < 0.001)

Since our findings indicate that accumulation of misfolded SOD1 results in a defective nuclear export (Fig. 1f, g), we examined the distribution of XPO1 in motor neurons from the lumbar spinal cord of mutant SOD1^G93A^ mice at different stages of the disease. XPO1, also referred to as chromosome region maintenance protein 1 (CRM1), regulates the export of cargoes, including proteins and several classes of RNAs, from the nucleus to the cytoplasm [55]. While XPO1 localized to the nucleus in 160-day-old non-transgenic mice, the protein gradually mislocalized to the nuclear membrane and the cytoplasm as disease progressed in mutant SOD1^G93A^ mice (Fig. 2a, b). Cytoplasmic accumulation led to a profound nuclear depletion of XPO1 by the end stage of the disease (Fig. 2a). Abnormal distribution of XPO1 was also observed in other SOD1-ALS mouse models, including the late onset SOD1^G37R^ (Fig. 2c–e) and dismutase inactive SOD1^G85R^ mice (Supplementary Fig. 2). Notably, nuclear depletion of XPO1 in the spinal cord was not accompanied by nuclear loss of TDP-43, a protein mislocalized in almost all cases of ALS except those carrying mutations in SOD1 and FUS [56, 57] (Fig. 2f, g).

The progressive decline in nuclear XPO1 levels is associated with the accumulation of misfolded SOD1 in the cytoplasm as disease progresses (Fig. 2a and Supplementary Fig. 3a-d) [53]. However, in mutant SOD1-expressing SH-SY5Y cells that accumulate misfolded SOD1, endogenous XPO1 was retained in the nucleus, suggesting that XPO1 mislocalization may be a consequence of prolonged, progressive stress that may not be manifested within the short time frame of in vitro cellular models (Supplementary Fig. 3e-g) [58–60].

Zhong and colleagues have previously reported that cytosolic accumulation of misfolded SOD1 results from the association between XPO1 and the NES-like sequence of the SOD1 protein exposed upon protein misfolding [52]. They showed that mutation (L38R) within the NES-like sequence disrupted the binding of XPO1 to SOD1^G93A^ and restored the normal distribution of SOD1 protein throughout the cell [52]. We examined whether dissociation of XPO1 from misfolded SOD1 would have a beneficial effect on the nuclear export defect. To this end, we generated constructs expressing SOD1^G93A^ with the L38R mutation to interrupt its binding to XPO1 (Supplementary Fig. 4a, b). Co-transfection of S-GFP and SOD1 mutants (SOD1^G93A^ or SOD1^G93A/L38R^) in SH-SY5Y cells, demonstrated that preventing the binding of mutant SOD1 to XPO1 restored the localization of S-GFP predominantly to the cytoplasm, as observed in cells expressing SOD1^WT^ (Supplementary Fig. 4c, d).

### XPO1 is localized into activated microglia in the lumbar spinal cord of mutant SOD1 mice

**Fig. 3:**
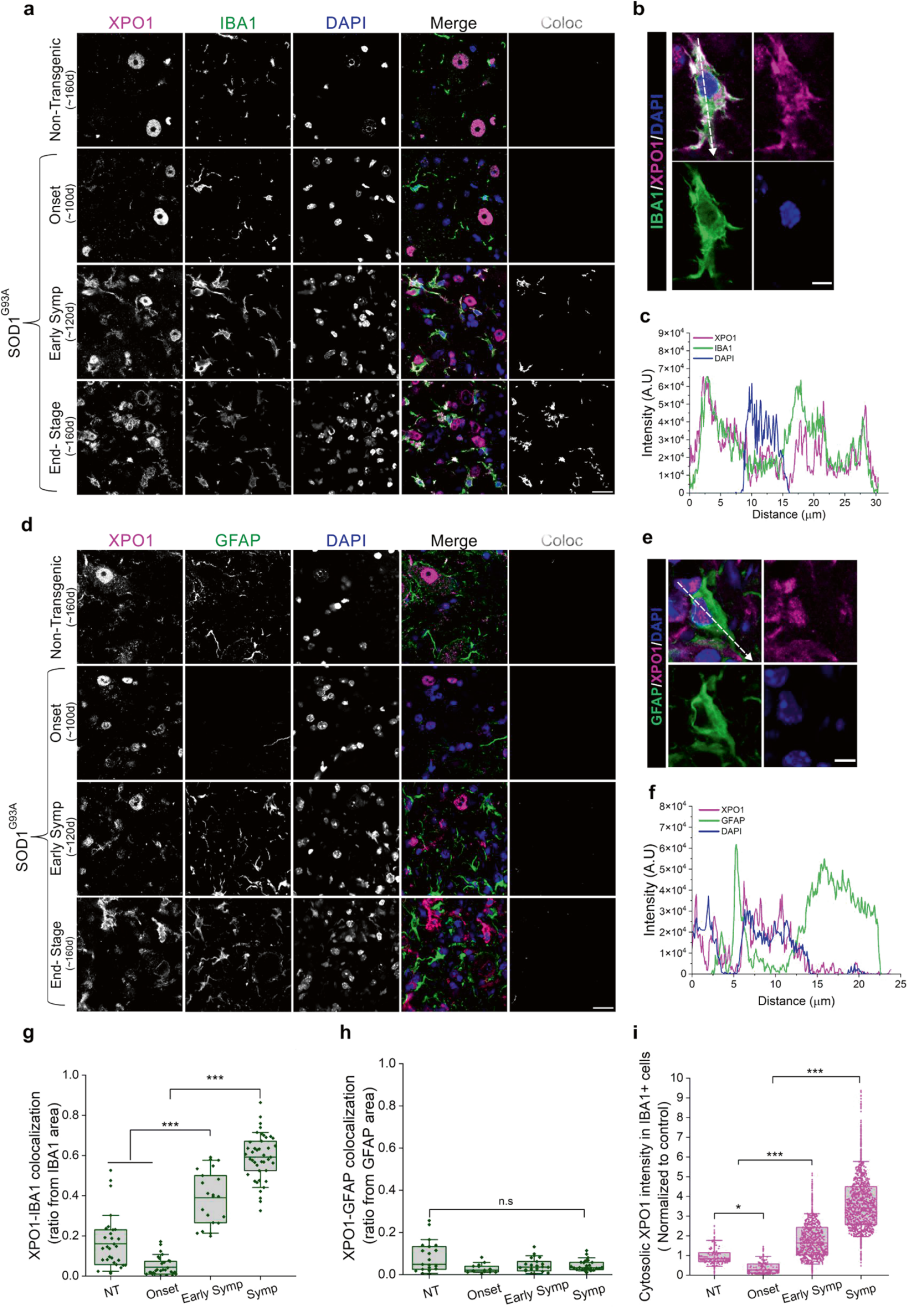
Exportin 1 accumulates in the cytoplasm of microglia in the spinal cord of mutant SOD1 mice. **a**. Immunofluorescent staining of lumbar spinal cord sections from non-transgenic (*n* = 3) and SOD1^G93A^ (*n* = 3) mice at different stages of the disease, with antibodies against XPO1 (magenta), Iba1 (green), and DAPI (blue). Scale bar, 20 µm. **b**, **c**. An intensity profile plot line (white dashed arrow) was drawn through Iba1-positive cells (**b**), and signal intensity was plotted across the length of the line to determine the co-localization of XPO1 and Iba1 proteins within microglia (**c**). Scale bar, 5 µm. **d**. Immunofluorescent staining of lumbar spinal cord sections from non-transgenic (*n* = 3) and SOD1^G93A^ (*n* = 3) mice at different stages of the disease, with antibodies against XPO1 (magenta), GFAP (green), and DAPI (blue). Scale bar, 20 µm. **e**, **f**. An intensity profile plot line (white dashed arrow) was drawn through GFAP-positive cells (**e**), and signal intensity was plotted across the length of the line to determine the co-localization of XPO1 and GFAP proteins within astrocytes (**f**). Scale bar, 5 µm. **g**. Quantification of Iba1 and XPO1 staining overlap in (**a**). **h**. Quantification of GFAP and XPO1 staining overlap in (**d**). **i**. Quantification of XPO1 intensity in microglia in spinal cord sections from non-transgenic (*n* = 3) and SOD1^G93A^ (*n* = 3) mice at different stages of the disease. Graphs represent quartiles (boxes), 50th percentiles (center lines) and range (10–90; whiskers). Three independent experiments for (**g**) and (**h**) (each dot represents a colocalization image. Rank-based one-way ANOVA, p-values adjusted for multiple comparisons and clustering; n.s. non-significant, ***p < 0.001), three independent experiments for (**i**) (dots represent quantified Iba1-positive microglia cells from all the experiments. Rank-based one-way ANOVA, p-values adjusted for multiple comparisons and clustering; *p < 0.05, ***p < 0.001)

We noticed that at early symptomatic stage (120-day), while XPO1 is not yet significantly depleted from the nuclei of motor neurons (Fig. 2b), it accumulated in the cytoplasm of cells surrounding spinal motor neurons of mutant SOD1^G93A^ mice (Supplementary Fig. 5a). Staining of the lumbar spinal cord of mutant SOD1^G93A^ mice at different stages of the disease with antibodies recognizing XPO1 and either ionized calcium-binding adaptor molecule 1 (Iba1) or glial fibrillary acidic protein (GFAP) revealed that expression of cytoplasmic XPO1 significantly increased in microglia after disease onset (Fig. 3). Indeed, XPO1 staining co-localized with the cytoplasmic Iba1 protein in microglia (Fig. 3a–c, g) but remained mostly nuclear in GFAP-positive astrocytes (Fig. 3d–f, h). In contrast, XPO1 was primarily localized within the nucleus of Iba1-positive cells at disease onset (100 days), suggesting that its cytosolic accumulation is associated with disease progression (Fig. 3i, Supplementary Fig. 5b-e). Moreover, we found XPO1 cytoplasmic accumulation in microglia in other SOD1-ALS models, with a significant colocalization between cytoplasmic Iba1 and XPO1 in symptomatic SOD1^G37R^ mice (Supplementary Fig. 6a, c) and a similar trend in SOD1^G85R^ mice (Supplementary Fig. 6b, d). Consistent with impairment of NCT being a hallmark of normal aging [35, 39], cytoplasmic localization of XPO1 in Iba1-positive microglia was also increased in 370-day-old non-transgenic mice (Supplementary Fig. 6) compared to 160-day-old animals (Fig. 3a, g). While Iba1 is considered a pan microglial marker [61], we and others [62–64] showed that its expression also correlated with microglial activation as revealed by their increase in number (Supplementary Fig. 6e) and the change in their morphology as disease progressed. Using staining for CD68, we confirmed the increase of XPO1 cytoplasmic accumulation in activated microglia in the spinal cord of end stage SOD1^G93A^ mice (Supplementary Fig. 7a-d).

Remarkably, cytoplasmic accumulation of XPO1 within microglia (Fig. 3a–c, i) did not result from increased expression, as indicated by the XPO1 RNA levels assessed by microarrays in microglia isolated from the lumbar spinal cord of symptomatic SOD1^G93A^ mice [65] (Supplementary Fig. 7e). Similarly, the expression of XPO1 mRNA in spinal motor neurons exhibited only slight elevation, as demonstrated by ribosome affinity purification coupled with high-throughput sequencing (BacTRAP) analysis of spinal motor neurons isolated from SOD1^G37R^ mice [66] (Supplementary Fig. 7f). This modest upregulation may represent a compensatory mechanism in response to the observed nuclear depletion of XPO1 in these motor neurons. Notably, in microglia, despite unchanged RNA levels, total XPO1 protein levels were elevated, suggesting impaired protein clearance within microglia or possible engulfment of XPO1 from neighboring cells (Supplementary Fig. 7 g, h).

### SOD1 silencing with virally delivered shRNA rescues XPO1 nuclear localization

**Fig. 4:**
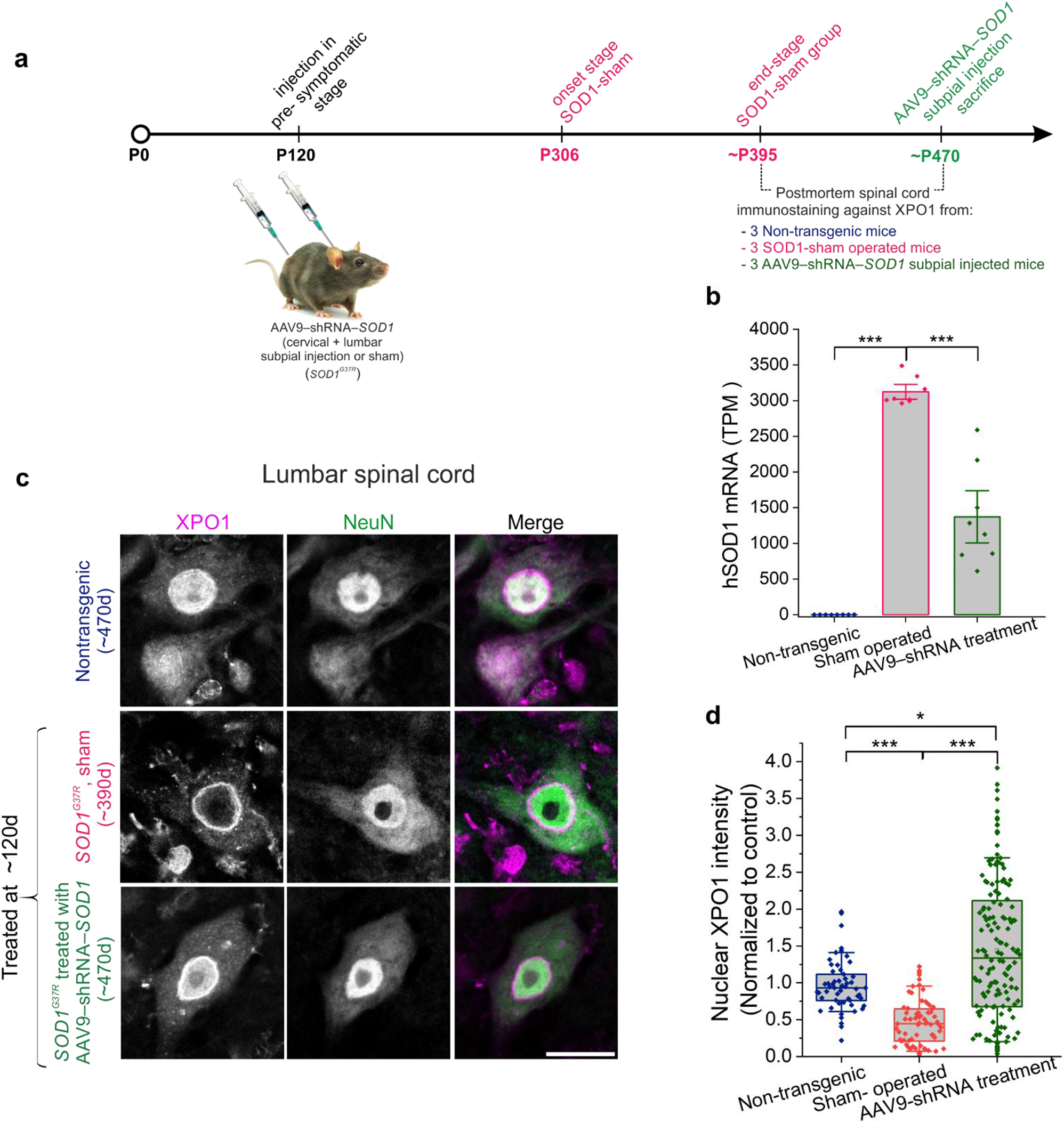
Mutant SOD1 silencing rescues XPO1 distribution in spinal motor neurons of mutant SOD1 mice. **a**. Schematic diagram of experimental design. SOD1^G37R^ mice received cervical and lumbar spinal subpial (SP) injections of AAV9–shRNA–SOD1 or AAV9-empty vector before disease onset (defined by a 20% decrease in grip strength and open-field motor performance). Post-mortem tissues were harvested for immunostaining. **b**. Quantification of hSOD1 mRNA in the lumbar and cervical spinal cord of subpial treated SOD1^G37R^ mice compared to control and sham-operated groups. (dots represent counts of hSOD1 mRNA from individual lumbar section of spinal cord. Mann-Whitney test; ***p < 0.001). **c**. Immunofluorescence of lumbar spinal cord sections from wild-type non-transgenic (*n* = 3), sham-operated SOD1^G37R^ (*n* = 3) and AAV9–shRNA–SOD1-treated SOD1^G37R^ (*n* = 3) mice stained against XPO1 (magenta) and NeuN (green); scale bar, 20 µm. **d**. Quantification of nuclear XPO1 intensity from (**c**). Graphs represent quartiles (boxes), 50th percentiles (center lines) and range (10–90; whiskers). Three independent experiments for (**c**) (dots represent individual neurons from all experiments. Rank-based one-way ANOVA, p-values adjusted for multiple comparisons and clustering; *p < 0.05, ***p < 0.001)

Bravo-Hernandez and colleagues recently developed a strategy to efficiently transduce spinal cord with AAV using spinal subpial injection at the cervical and lumbar levels [64]. Delivery of AAV9 encoding an shRNA against SOD1 into the spinal cord of adult presymptomatic SOD1^G37R^ mice reduced accumulation of SOD1 and suppressed motor neuron disease (Fig. 4a, b) [64]. We determined the cellular localization of XPO1 by immunostaining of spinal cord tissues from non-transgenic mice, SOD1^G37R^ sham operated mice and SOD1^G37R^ mice subpially treated with AAV9–shRNA–SOD1. XPO1 accumulated at the nuclear membrane in SOD1^G37R^ sham operated mice (Fig. 4c), with a significant decrease of nuclear XPO1 intensity in Sham-operated SOD1 mice compared to non-transgenic animals (Fig. 4c, d). The nuclear localization of XPO1 was significantly restored in transgenic mice that received spinal subpial injection of AAV9–shRNA–SOD1 compared to Sham-operated SOD1 mice. The nuclear intensity of XPO1 showed variability between individual motor neurons with a subset of neurons accumulating higher levels than in non-transgenic animals, possibly reflecting an excessive correction of XPO1 nuclear localization upon depletion of mutant SOD1 (Fig. 4c, d and Supplementary Fig. 7i).

### Mutant SOD1 alters the distribution and density of nucleocytoplasmic import factors

**Fig. 5:**
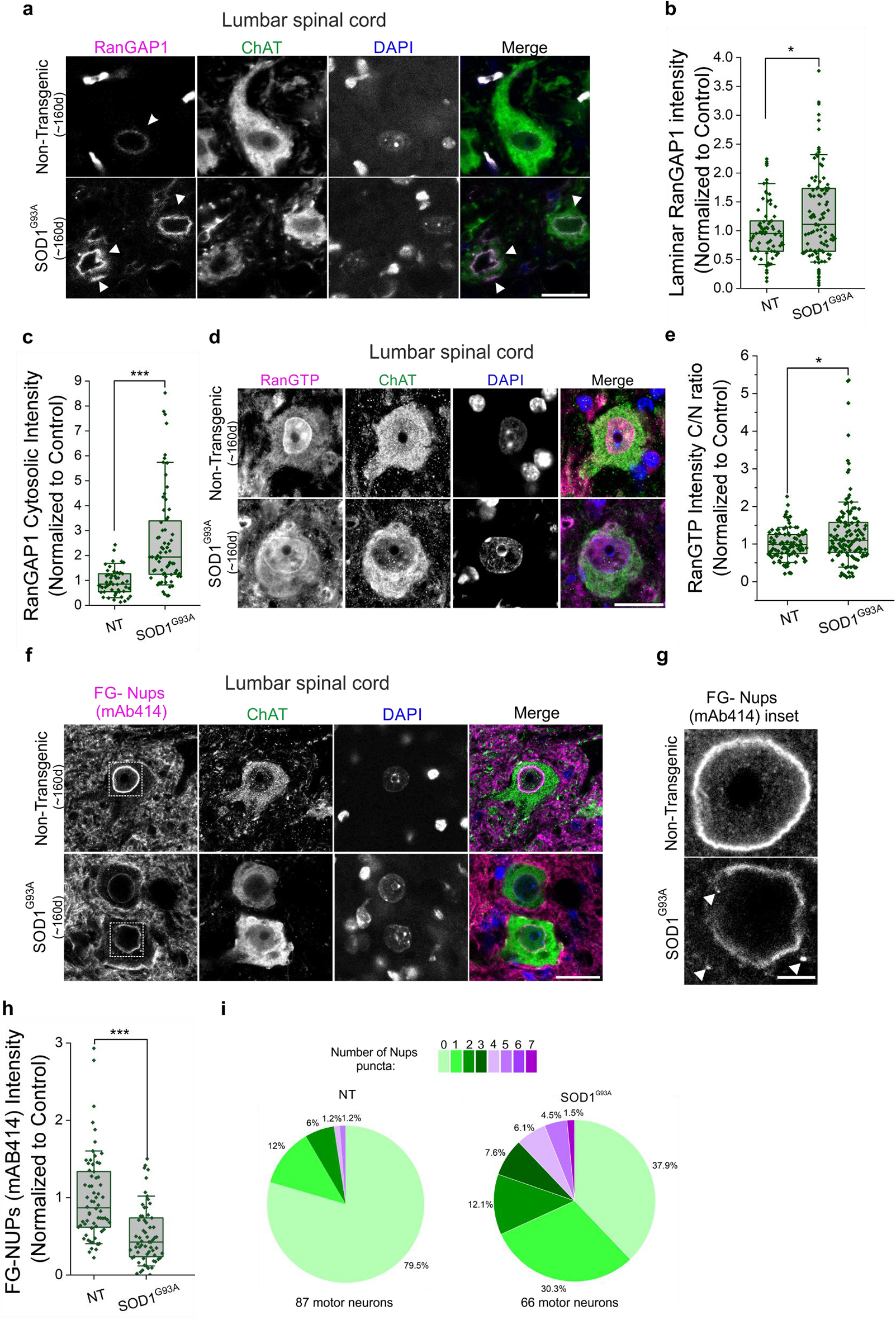
SOD1 pathology is associated with disrupted import transport factors and decreased NPC density. **a**. Immunofluorescence of endogenous RanGAP1 (magenta) in ChAT-positive cells (green) from lumbar spinal cord sections of non-transgenic (*n* = 4) and end-stage SOD1^G93A^ (*n* = 4) mice. DAPI (blue) was used to detect the nucleus; scale bar, 20 µm. **b**. Quantification of the laminar intensity of RanGAP1 represented in (**a**). **c**. Quantification of the cytosolic distribution of RanGAP1 in spinal motor neurons represented in (**a**). **d**. Immunofluorescence of endogenous RanGTP (magenta) in ChAT-positive cells (green) from lumbar spinal cord sections of non-transgenic (*n* = 3) and end-stage SOD1^G93A^ (*n* = 4) mice. **e**. Quantification of the RanGTP gradient in spinal motor neurons from staining in (**d**). **f**. Immunofluorescence of endogenous FG-Nups (recognized with mAb414 antibody ) (magenta) in ChAT-positive cells (green) from lumbar spinal cord sections of non-transgenic (*n* = 3) and end-stage SOD1^G93A^ (*n* = 3) mice. **g**. Inset of the motor neurons nuclear pore complex staining from (**f**) highlighting the diminished staining intensity and the presence of FG-Nups inclusions in spinal cords of SOD1^G93A^ mice compared to control; scale bar, 5 µm **h**. Quantification of the laminar intensity of FG-Nups represented in (**f**). **i**. Pie chart of the number of motor neurons with puncta in (**f**). Graphs represent quartiles (boxes), 50th percentiles (center lines) and range (10–90; whiskers). Four independent experiments for (**b**) and (**c**) (dots represent quantified motor neurons from all experiments. Rank-based two-samples t-test, p-values adjusted for clustering; *p < 0.05, ***p < 0.001), three independent experiments for e (dots represent quantified motor neurons from all experiments. Rank-based two-samples t-test, p-values adjusted for clustering; *p < 0.05), three independent experiments for (**h**) (dots represent quantified motor neurons from all experiments. Rank-based two-samples t-test, p-values adjusted for clustering; ***p < 0.001)

Based on previous studies reporting deficits in nuclear import in neurodegenerative disorders [15, 17, 34–36, 67, 68] and particularly ALS [18, 19, 21, 23, 27, 42, 69, 70], we proceeded to examine proteins critical for functional nuclear import in SOD1^G93A^ mice. During protein import, cargo is released into the nucleus when the import receptor interacts with Ran-GTP, a GTP-binding nuclear protein. During nuclear export, cargo is released into the cytoplasm upon GTP hydrolysis of Ran-GTP by RanGAP1, a GTPase-activating protein located on the cytoplasmic filaments of the NPC. Higher levels of Ran-GTP in the nucleus than in the cytosol (Ran gradient) and its maintenance by RanGAP1 is critical for sustained active transport through the NPC [71]. Notably, we observed elevated RanGAP1 intensity at the nuclear envelope in mutant SOD1-expressing SH-SY5Y cells compared to those expressing wild-type SOD1 (Supplementary Fig. 8a, b). Therefore, we examined the distribution of RanGAP1 in the lumbar spinal cord of symptomatic mutant SOD1^G93A^ mice and non-transgenic littermates. While RanGAP1 surrounded the nuclear envelope in a thin ring-shaped manner in non-transgenic mice, its intensity was increased leading to a thicker ring surrounding the nuclear envelope (Fig. 5a, b and Supplementary Fig. 8c) and cytosolic accumulation (Fig. 5a, c) in SOD1^G93A^ mice. In addition, a slight shift of the Ran-gradient towards the cytosol was detected in spinal motor neurons of SOD1^G93A^ mice compared to their non-transgenic littermates (Fig. 5d, e and Supplementary Fig. 8d), suggesting altered nuclear import in mutant SOD1 mice.

Given the critical role of RanGAP1 in protein import, and its well-documented dysregulation in neurodegenerative diseases [19, 20, 34–36, 69], we next examined its distribution in microglial cells, analogous to our analysis of XPO1. RanGAP1 displayed a comparable pattern of cytosolic accumulation, with marked colocalization with cytoplasmic Iba1 (Supplementary Fig. 9a-d). Importantly, this pattern was not observed when examining TDP-43 in microglial cells, as it remained predominantly nuclear (Supplementary Fig. 9e-h). This suggests that the disruption observed in microglia is specific to a subset of proteins involved in NCT, rather than a generalized disruption of nuclear import. Notably, despite mislocalization of XPO1 (Fig. 3) and RanGAP1 (Supplementary Fig. 9a-d) in microglia, colocalization analysis did not reveal the presence of misfolded SOD1 in Iba1-positive cells. The mean ratio of the overlapping area between Iba1 and B8H10 signals relative to the total Iba1-positive area was 0.035, indicating a very low level of colocalization and the absence of misfolded SOD1 in these cells (Supplementary Fig. 10a-c). Furthermore, immunostaining of primary cultured microglia using the B8H10 antibody for misfolded SOD1 did not detect any signal (Supplementary Fig. 10d).

Nucleocytoplasmic translocation requires that nuclear transport receptors (together with RanGTP/RanGDP) bind low-complexity domains of FG-Nups that constitute the hydro-gel barrier of the nuclear pore [72]. We analyzed ChAT-positive cells of the lumbar spinal cord of non-transgenic and symptomatic SOD1^G93A^ mice stained with an antibody (mAb414) recognizing nucleoporins of the FG-Nup family (i.e., Nup62, Nup153, Nup214, and Nup358). Motor neurons of non-transgenic mice displayed an intense FG-Nup staining around the nucleus. In contrast, mutant SOD1^G93A^ motor neurons showed a significantly reduced staining of FG-Nups at the nuclear envelope (Fig. 5f–h and Supplementary Fig. 11a). In addition, inclusions of FG-Nups (indicated with white arrows) were significantly increased in motor neurons of SOD1^G93A^ mice compared to non-transgenic littermates (Fig. 5g, i). Consistent with the marked reduction seen in mutant SOD1^G93A^ motor neurons from transgenic mice, SH-SY5Y cells expressing mutant SOD1^G93A^ exhibited a significant decrease in FG-Nups levels (Supplementary Fig. 11b, c). These findings demonstrate that mutant SOD1 disrupts crucial components of the NCT system and vital nucleoporins as the disease progresses.

### SOD1 mutation leads to increased membrane discontinuity and fragmentation

FG-Nup staining in ChAT-positive motor neurons from the lumbar spinal cords of non-transgenic and end-stage (160-day-old) SOD1^G93A^ mice did not indicate abnormal nuclear circularity in SOD1^G93A^ motor neurons (Supplementary Fig. 12a, b). However, while control neurons typically displayed a robust and continuous FG-Nup signal along the nuclear membrane, motor neurons from SOD1^G93A^ mice frequently exhibited discontinuous FG-Nup labeling, with multiple gaps observed around the nuclear envelope (Supplementary Fig. 12a, c–f). These discontinuities resulted in an increased number of FG-Nup fragments in mutant SOD1 expressing nuclei compared to control nuclei (Supplementary Fig. 12e).

To further characterize this disruption, we calculated a continuity index—defined as the ratio of the total length of fragmented membrane signal to the nuclear perimeter—where lower values indicate greater discontinuity. This analysis showed that motor neurons nuclei from SOD1^G93A^ mice had significantly lower continuity indices than controls (Supplementary Fig. 12f).

Together, these results indicate that although nuclear shape remains largely preserved in mutant SOD1-ALS motor neurons, the structural integrity of the nuclear membrane is compromised, as evidenced by fragmented and discontinuous FG-Nup staining.

### Nucleocytoplasmic transport disruption in SOD1-ALS patients derived fibroblasts

**Fig. 6:**
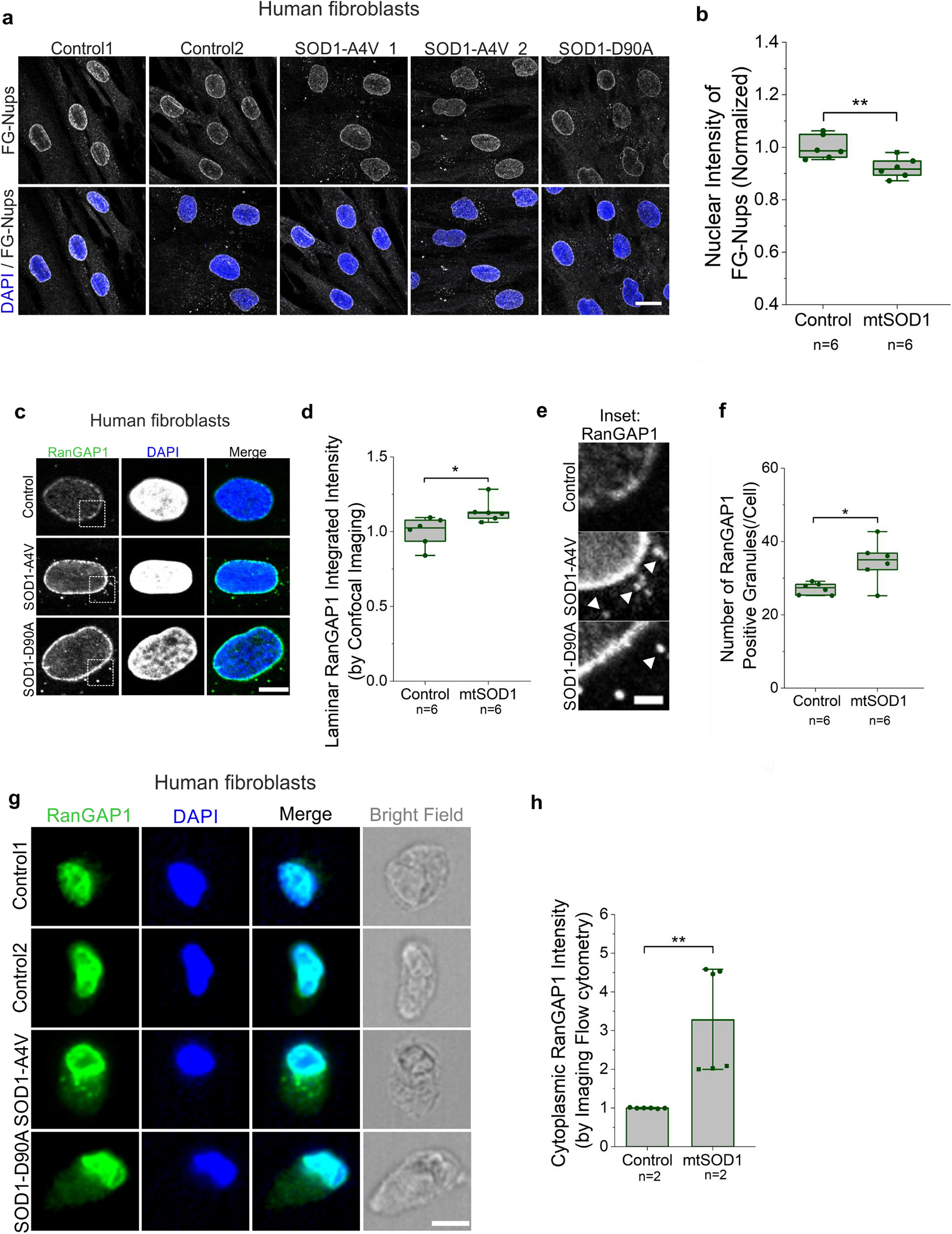
Nucleocytoplasmic transport impairment in fibroblasts carrying SOD1 mutation. **a**. Representative confocal images of immortalized fibroblasts from SOD1-ALS patients and healthy controls immunostained for FG-Nups (gray) and counterstained with DAPI (blue). Scale bar, 20 µm. **b**. Quantification of nuclear FG-Nups intensity in six mutant SOD1-ALS fibroblasts (five SOD1–A4V, one SOD1–D90A) and six controls. The box plot reflects median per well; each data point indicates the average for each line from two independent experiments. Statistical significance was assessed across data points. Unpaired t-test; **p < 0.01. **c**. Confocal imaging after immunofluorescence staining of RanGAP1 (green) and DAPI staining (blue). Scale bar, 10 $\mu$m. **d**. Quantification of RanGAP1 accumulation at the nuclear membrane (laminar intensity) from six healthy controls and six SOD1-ALS patients (five SOD1–A4V and one SOD1–D90A) in (**c**). The box plot reflects the median per well; each data point indicates the average per line. Statistical significance was assessed across data points. Unpaired t-test; *p < 0.05. **e**. Inset of the fibroblasts RanGAP1 staining from (**c**) highlighting the presence of RanGAP1 inclusions in fibroblasts carrying SOD1 mutants (A4V, D90A) compared to control; scale bar, 3 µm. **f**. Quantification of RanGAP1 cytoplasmic puncta in fibroblasts using confocal imaging. The box plot reflects the median per well, and each data point indicates the average for each line from three individual experiments. Statistical significance was assessed across data points. Unpaired t-test; *p < 0.05. g. Flow cytometry-based imaging of fibroblasts stained for RanGAP1 (green) and DAPI (blue). Bright field is shown in gray. Scale bar, 10 $\mu$m. **h**. Flow cytometry-based imaging analysis of cytosolic RanGAP1 intensity in fibroblasts from two healthy controls, one SOD1–A4V, and one SOD1–D90A ALS patients. Each dot represents the average from a single experiment (three experiments per line); squares indicate data from the SOD1–D90A line. Statistical analysis was performed using an unpaired t-test comparing the experimental averages for each line; **p < 0.01

We next investigated nucleocytoplasmic transport factors in fibroblasts from ALS patients carrying different SOD1 mutations (A4V and D90A). Consistent with our results in mice, confocal imaging showed a significant reduction of FG-Nups staining in immortalized SOD1-ALS fibroblasts compared to control fibroblasts (Fig. 6a, b). We also observed a significant increase of RanGAP1 at the nuclear envelope in SOD1-ALS patient fibroblasts compared to controls (Fig. 6c, d). In addition, the number of RanGAP1 inclusions surrounding the nuclei was increased in SOD1–A4V and D90A mutant fibroblasts compared to controls (Fig. 6e, f), without a significant change in their size (Supplementary Fig. 13a). We then utilized flow cytometry-based imaging to quantify morphological and spatial features in thousands of cells per condition [73, 74]. Following RanGAP1 immunostaining of fibroblasts in suspension, more than 10,000 cells per line were analyzed for RanGAP1 cytoplasmic localization. Fibroblasts with SOD1–D90A and SOD1–A4V mutations displayed significantly increased cytosolic RanGAP1 when compared to controls (Fig. 6g, h).

In contrast, no significant differences in cytosol-to-nucleus (C/N) ratios were observed between control and SOD1 mutant fibroblasts for RanGTP (Supplementary Fig. 13b, c), XPO1 (Supplementary Fig. 13d, e) or TDP-43 (Supplementary Fig. 13f, g). In addition, assessment of the nuclear envelope morphology using Lamin B1 immunostaining (Supplementary Fig. 14a, b) and flow cytometry-based imaging (Supplementary Fig. 14c-e) did not reveal nuclear shape distortion or aberrant Lamin B1 localization in mutant SOD1 fibroblasts compared to healthy controls. Notably, in accordance with the limited alterations observed in patient fibroblasts, we did not detect accumulation of misfolded SOD1 in this cellular model (Supplementary Fig. 14f).

### Impaired nucleocytoplasmic compartmentalization in the spinal cord of SOD1-ALS patients

**Fig. 7:**
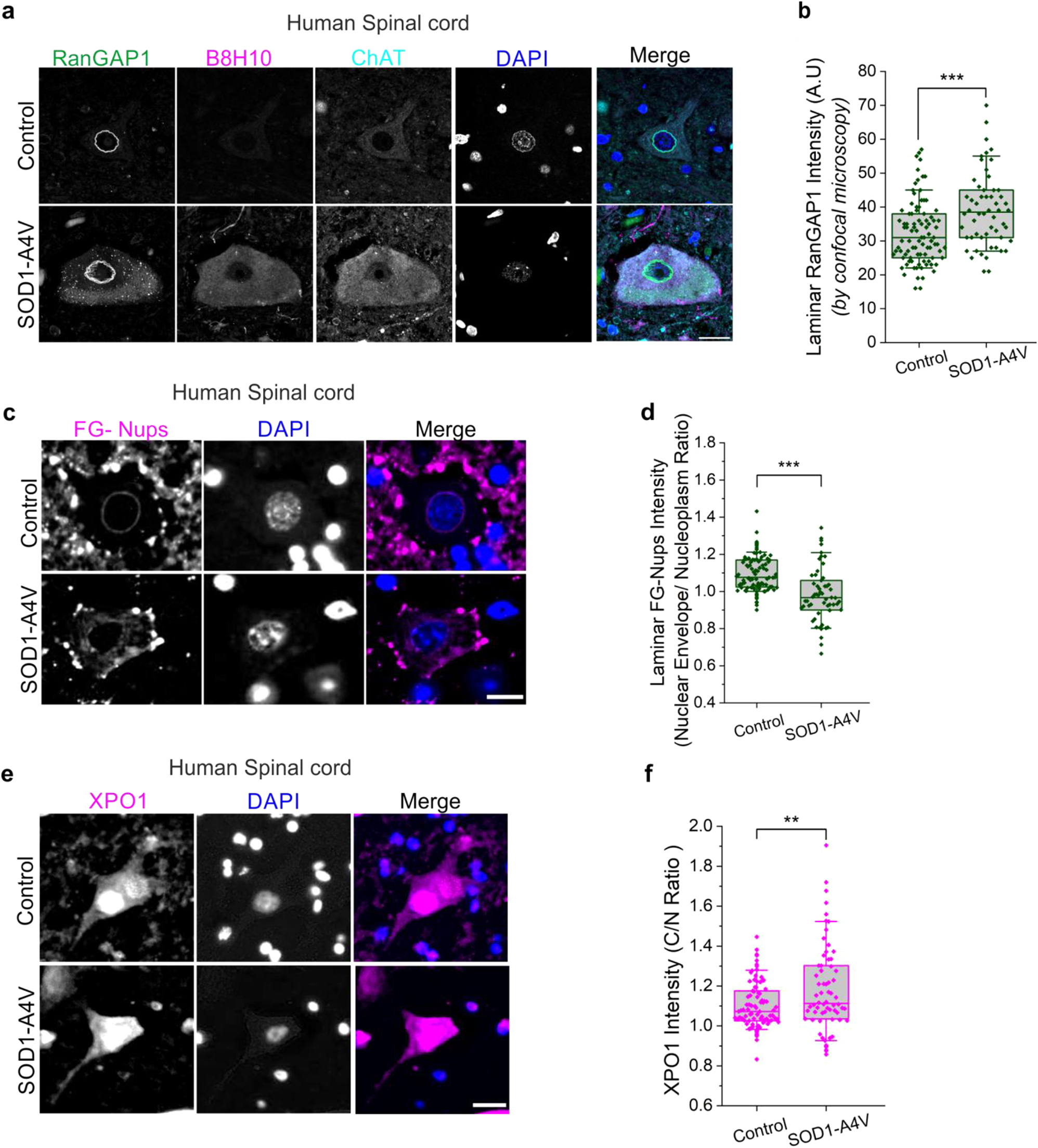
Nucleocytoplasmic transport disruption in SOD1-ALS postmortem spinal cord tissues. **a**. Confocal imaging after immunofluorescence staining of RanGAP1 (green), B8H10 for misfolded SOD1 (magenta), ChAT (cyan), and DAPI (blue) in postmortem lumbar spinal cord from a control individual and a patient with SOD1^A4V^ mutation. Scale bar, 50 $\mu$m. **b**. Quantification of RanGAP1 accumulation at the nuclear membrane (laminar intensity) in lumbar spinal cords from controls (*n* = 5) and SOD1^A4V^ ALS patients (*n* = 4). **c**. Confocal imaging after immunofluorescence staining of FG-Nups (magenta) and DAPI (blue) in postmortem cervical spinal cord from a control individual and a patient carrying SOD1^A4V^ mutation. Scale bar, 20 $\mu$m. **d**. Quantification of FG-Nups intensity at the nuclear membrane as the ratio of nuclear envelope to nucleoplasm signal intensity in cervical spinal cord sections from controls (*n* = 3) and SOD1^A4V^ ALS patients (*n* = 3). **e**. Confocal imaging after immunofluorescence staining of XPO1 (magenta) and DAPI (blue) in postmortem cervical spinal cord from a control individual and a patient carrying SOD1^A4V^ mutation. Scale bar, 20 $\mu$m. **f**. Quantification of cytosol-to-nucleus ratio of XPO1 signal intensity in cervical spinal cord sections from controls (*n* = 3) and SOD1^A4V^ ALS patients (*n* = 3). Graphs represent quartiles (boxes), 50th percentiles (center lines) and range (10–90; whiskers. For (**b**) data were quantified from 5 healthy controls and 4 SOD1^A4V^ ALS patients (dots represent quantified motor neurons from all experiments. Rank-based two-samples t-test, p-values adjusted for clustering; ***p < 0.001). For (**d**) and (**f**), data were quantified from 3 healthy controls and 3 SOD1^A4V^ ALS patients (dots represent quantified motor neurons from all experiments. Unpaired parametric t-test, **p < 0.01, ***p < 0.001)

To further validate our findings in SOD1-ALS patients, we performed staining of motor neurons from the lumbar and cervical spinal cords of postmortem tissues from three individuals carrying the SOD1–A4V mutation. This analysis revealed altered expression and distribution of RanGAP1 in SOD1-ALS cases compared to age-matched controls, using two distinct microscopy imaging techniques (Fig. 7a, b and Supplementary Fig. 15a, b). In addition, immunofluorescence staining of FG-Nups revealed a clear distinction in nuclear pore complex (NPC) distribution. Control samples showed strong, consistent perinuclear FG-Nup enrichment, reflecting preserved nuclear envelope integrity. In contrast, SOD1–A4V ALS tissues exhibited a marked reduction in FG-Nup signal at the nuclear rim, indicating possible disorganization of the nuclear envelope or reduced NPC expression (Fig. 7c, d). Moreover, while XPO1 was predominantly localized within the nucleoplasm in control neurons, neurons from SOD1–A4V ALS displayed prominent cytoplasmic mislocalization of XPO1 (Fig. 7e, f). Collectively, these findings reinforce nucleocytoplasmic transport disruption as a pathological hallmark of SOD1-associated ALS.

## Discussion

Numerous cellular pathways have been proposed to contribute to mutant SOD1-associated familial ALS, including excitotoxicity [75, 76], ER stress [77, 78], mitochondrial dysfunction [79–81], axonal-transport dysfunction [82, 83] and prion-like propagation [84, 85]. Disruption of nucleocytoplasmic transport has emerged as a core mechanism in ALS pathology as well as several other neurodegenerative disorders but had not been thoroughly investigated in SOD1-ALS. Using in vitro and in vivo models, we demonstrate that misfolded SOD1 leads to severe impairment of NCT marked by (1) altered distribution of a nuclear transport reporter; (2) loss of crucial Nups from the nuclear pore; (3) disruption of the Ran-GTP gradient; (4) abnormal accumulation and localization of the GTPase-activating protein RanGAP1; and (5) mislocalization of the karyopherin family member, XPO1, in motor neurons and microglia. Importantly, we showed restoration of XPO1 nuclear depletion in spinal cord samples from SOD1^G37R^ mice upon delivery of an shRNA–SOD1-silencing vector [64], supporting a causal relationship between accumulation of misfolded SOD1 and NCT impairment. Notably, defects in import and export factors were also observed in human fibroblasts and postmortem spinal cord tissues from SOD1-ALS patients. Together, our results indicate that expression of mutant SOD1 substantially affects both the import and export of nuclear protein trafficking. These results are consistent with previous reports in other ALS forms with mutations in C9orf72 [19–21, 24, 26], TDP-43 [22], PFN1 [23], FUS [27] and sporadic ALS [22, 26, 86, 87], further supporting disruption of NCT as a common mechanism in ALS pathogenesis.

It was proposed that cytoplasmic but not nuclear artificial β-sheet aggregates trigger mislocalization of nuclear transport receptors and inhibition of RNA export [14]. Consistently, we found that cytosolic accumulation of misfolded SOD1 during disease progression correlates with the disruption of NCT; however, the mechanisms by which accumulation of misfolded SOD1 leads to alteration of nuclear import and export regulators remains to be explored. Notably, previous in vitro studies identified a direct interaction between SOD1, in its misfolded form, and the nuclear transport receptor XPO1 as the cause for the cytosolic accumulation of misfolded SOD1 [52]. In this study, we demonstrate that XPO1 is depleted from the nucleus and accumulates in the cytosol of motor neurons in three different mutant SOD1 transgenic mouse models and in postmortem tissues from ALS patients carrying SOD1–A4V mutation. Among the six exportin family members, XPO1 has been associated with the occurrence of aberrant nuclear export in several neurological diseases, including traumatic brain injury [88], Alzheimer’s disease [36, 68], Huntington’s disease [14, 34, 35], ALS [19, 20, 22, 89] and multiple sclerosis [90, 91]. Notably, while XPO1 was found mislocalized in mouse and patient tissues, it was not altered in SH-SY5Y cells expressing mutant SOD1, nor in patient fibroblasts, suggesting that XPO1 mislocalization is a consequence of prolonged, progressive stress that may not be manifested within the short time frame of in vitro disease models.

By leveraging tissues from a previously published study [64], we found that AAV-mediated silencing of mutant SOD1 restored nuclear localization of XPO1 levels in SOD1^G37R^ mice. Notably, a subset of neurons from SOD1^G37R^ mice injected with AAV-shRNA accumulated higher levels of XPO1 than neuron from non-transgenic animals. This increase may reflect an excessive correction of XPO1 nuclear localization upon depletion of mutant SOD1. However, we cannot exclude that XPO1 levels are influenced by transduction of AAV since the control animals underwent surgical manipulation without AAV injection. We note previous transcriptomic analysis from these mice did not identify significant changes in XPO1 mRNA levels across treatment groups, indicating that the slight increase in nuclear XPO1 is not due to transcriptional activation [64].

While TDP-43 mislocalization represents a hallmark of most ALS subtypes, its presence in SOD1-linked ALS remains inconsistent across models. Although TDP-43 mislocalization was reported in mutant SOD1^G93A^ mice at disease end-stage [58], another study found preserved nuclear TDP-43 in spinal motor neurons of SOD1^G93A^, SOD1^G37R^ and SOD1^G85R^ mice [59]. Moreover, the absence of TDP-43 mislocalization in primary fibroblasts and postmortem tissues from SOD1–A4V patients has been previously described [22, 60]. These discrepancies may reflect differences in the specific SOD1 mutations, cell types analyzed, disease stages, or experimental conditions. Our findings align with the latter, showing TDP-43 nuclear localization in both SOD1–A4V and SOD1–D90A patient fibroblasts. In addition, our observation that TDP-43 is retained in the nucleus despite XPO1 cytoplasmic mislocalization in SOD1 transgenic mice is consistent with earlier studies showing that TDP-43 does not require XPO1 for export trafficking [92].

Previous observations regarding the disruption of NCT in different ALS models show low nuclear import rate [20–23, 42, 86, 93–95]. Here we observe that Ran-GTP and RanGAP1 are also disrupted in mutant SOD1 mice and patient fibroblasts. The mechanisms by which misfolded SOD1 impacts these two crucial proteins remain to be elucidated. While studies in C9orf72-ALS have demonstrated cytoplasmic mislocalization and depletion of RanGAP1 from the nuclear envelope [19], research in mutant SOD1 mice [95], sporadic ALS patients [95] and Huntington’s disease [34, 35] identified an age-dependent increase in nuclear envelope-associated RanGAP1, consistent with our findings.

Furthermore, we show that XPO1 and RanGAP1, but not TDP-43, accumulate not only in the cytosol of motor neurons but also in microglia in the lumbar spinal cord of symptomatic mutant SOD1 mice. Depending on the surrounding milieu, microglia can adopt either a resting or an activated state in response to threats, undergoing morphological changes, altered gene expression, and functional adjustments. In CNS injury and neurodegenerative diseases, they perform phagocytosis, clearing dead cells, antigens, and protein aggregates [96, 97]. The observed increased levels of XPO1 protein in microglia without a corresponding increase in RNA levels, suggests that cytosolic accumulation of XPO1 in microglia could stem either from its engulfment by microglia surrounding degenerating motor neurons or from XPO1 mislocalization and a clearance deficit within the microglia themselves.

Notably, we detected microglial XPO1 mislocalization in the absence of detectable misfolded SOD1 within these cells, indicating that XPO1 dysregulation in microglia may occur independently of misfolded SOD1 accumulation, thereby reinforcing the concept of non–cell-autonomous mechanisms. Consistent with this concept, cytosolic XPO1 was also detected in microglia within the spinal cord of aging wild-type mice consistent with previous reports of age-related disruption of the nuclear pores and NCT, as a contributing factor to neurodegenerative disease pathogenesis [39, 98]. 

Together, these findings suggest that misfolded SOD1 accumulation and aging-associated NCT alterations may contribute to a deleterious microenvironment that could propagate neurodegeneration through both cell-autonomous and non–cell-autonomous mechanisms.

In addition, FG-Nup staining revealed frequent discontinuities in SOD1^G93A^ motor neurons, leading to increased FG-Nup fragmentation and reduced nuclear envelope continuity along with decreased signal and punctuation. These findings point to selective disruption of NPC components in the absence of gross nuclear deformation. Notably, our observations are consistent with previous work by Kinoshita and colleagues, who reported irregular nuclear contours and disrupted Nup62 localization in anterior horn cells from ALS patients and SOD1 mutant mice [86], reinforcing the notion that NCT deficits in ALS stem also from progressive alterations at the nuclear pore level.

An indirect effect of oxidative stress may explain the NCT impairment caused by misfolded SOD1 revealed in this study [99]. Indeed, mutant SOD1 was found to alter mitochondrial function in several studies resulting in oxidative stress [81, 100–103] which has been shown previously to cause the cytoplasmic mislocalization of nuclear proteins (including Ran), nuclear retention of importins, alteration of XPO1-dependent nuclear export, and reduced levels of several Nups [104–106]. Our study suggests a “domino effect” model in which mutant SOD1 leads to disruption of the nucleocytoplasmic transport both in neurons and microglia. Misfolded SOD1 leads to the mislocalization of nuclear transport regulators and Nups either by direct sequestration of these proteins by cytosolic misfolded SOD1 or indirectly from SOD1-mediated oxidative stress. It is possible that impairment of the NCT in microglial cells hinders their optimal functionality and indirectly affects the well-being of neurons that rely on the support provided by microglia. Taken together, our observations in SOD1-ALS models and SOD1 patients, along with overwhelming evidence in other ALS forms and neurodegenerative disorders, establishes disruption of the nuclear-cytoplasmic machinery as a central mechanism and significant target for therapeutic development. Although our study does not directly test the effects of restoring NCT function, previous studies in other neurodegenerative models have demonstrated that enhancing nuclear import or inhibiting nuclear export can confer neuroprotection [19, 20, 22, 29]. Thus, therapeutic strategies aimed at correcting NCT defects—either by stabilizing nuclear transport components or limiting misfolded SOD1 interactions—warrant further investigation in the context of SOD1-mediated ALS.

## Materials and Methods

### Plasmids

PcDNA 3.1 Shuttle-GFP was kindly provided by Prof. Dr. Franz-Ulrich Hartl from the Max-Planck-Institute for Biochemistry. pCI-hSOD1^WT^, pCIhSOD1^G93A^, and pCI-hSOD1^G85R^ were generated by inserting human SOD1 constructs into the pCI-NEO vector (Promega) between the EcoRI and the NotI sites. The double mutant SOD1^G93A/L38R^ was generated using a one-step PCR protocol with the pCIhSOD1^G93A^ as a template using the following primers to insert the L38R mutation:

Fwd: 5‘GGAAGCATTAAAGGACGGACTGAAGGCCTGCATG 3’

Rev: 5‘CATGCAGGCCTTCAGTCCGTCCTTTAATGCTTCC 3’

### Cell culture and transfection

Primary fibroblasts from six healthy control subjects (ND29510 and ND36320 from Coriell and Kin1ALS17, Kin1ALS6, Kin2ALS6, and Kin4ALS6 from UCSD) and six SOD1-ALS patients (ND29149, ND39022, and ND39023 from Coriell and ALS21, ALS22, and ALS45 from UCSD) were immortalized via hTERT transduction (Addgene). Fibroblasts were cultured from skin biopsies obtained after informed consent for this purpose and transferred by material transfer agreements. The cells were grown in high-glucose DMEM/F12 (Thermofisher Scientific) supplemented with 20% (vol/vol) fetal bovine serum (FBS) (Sigma), 1% (vol/vol) penicillin/streptomycin (Thermofisher Scientific). Human-derived neuronal-like cells (SH-SY5Y cells) were cultured in DMEM media (Biological Industries, Kibbutz Beit Haemek Israel) containing 10% FBS in a sterile incubator (Thermo Scientific) with 5% CO_2_ at 37 °C. For transfection, cells were cultured in 24 well plates on coverslips pre-sterilized with 70% ethanol and UV light. Transfection was performed using a TurboFect transfection reagent (Thermo Scientific, Inc, Lithuania) according to the manufacturer’s instructions, and transfected cells were incubated at 5% CO_2_ at 37 °C for 36 hours, followed by analysis. To confirm successful overexpression of mutant SOD1 by immunoblot, cells were simultaneously cultured in 35 mm dishes alongside the 24-well plates and transfected using the same transfection protocol as used for the immunofluorescence experiments.

### Primary cell culture (microglia)

Tissue Processing and Dissociation.

Spinal cords were extracted by hydraulic extrusion and immediately transferred into Hanks’ Balanced Salt Solution (HBSS; Sartorius, Germany) on ice to preserve tissue integrity. To dissociate the spinal cord tissue into single-cell suspensions, the Neural Tissue Dissociation Kit (P) (Miltenyi Biotec, Germany) was used according to the manufacturer’s instructions, with minor modifications. Specifically, to enhance tissue dissociation efficiency, a mechanical dissociation step was incorporated: tissue was triturated using a fire-polished glass pipette followed by filtration through a 70-μm cell strainer (Falcon) to remove debris and large undigested fragments.

### Cell plating and culture

The resulting cell suspension was plated onto culture plates pre-coated with poly-D-lysine (Sigma-Aldrich) and maintained in astrocyte growth medium under standard cell culture conditions (37 °C, 5% CO₂). Cells were cultured for 7 days to allow for adherence and selective expansion of glial populations.

### Cell replating and fixation

After the initial 7-day culture period, cells were detached using gentle enzymatic dissociation and replated into chamber slides for immunocytochemistry (ICC). Following 48 hours of additional culture, cells were fixed with 4% paraformaldehyde (PFA) for 15 minutes at room temperature and processed for immunostaining.

### Immunocytochemistry for primary culture

Fixed cells were permeabilized and blocked in a blocking solution with 10% donkey serum in PBS. Primary antibodies (Table 1) were applied overnight at 4 °C. Nuclei were counterstained with DAPI. Appropriate fluorescent secondary antibodies were used for detection, and samples were imaged using a Zeiss LSM880 confocal microscope (Carl Zeiss, Germany)

### Immunoblotting

Cell pellets were homogenized in an ice-cold lysis buffer [1X PBS, 0.2% Triton-X] containing a protease inhibitor cocktail (11836145001, ApexBio, 1:100) using a high speed 400 rpm Homogenizer (Glas-Col), centrifuged at 15,000*g* for 5 min at 4 °C, and the supernatant was transferred to a clean tube. Protein concentration was measured by Bradford method using bovine serum albumin (BSA) as standard. The samples (5–30 µg protein) were separated by a denaturing 14% Bis-Tris PAGE, and proteins transferred to a polyvinylidene difluoride (PVDF) membrane (Merck Millipore). The membrane was incubated for 1 hour at room temperature in a blocking buffer [5% (w/v) fat-free milk, 150 mM NaCl, 0.05% (v/v) Tween-20 in 20 mM Tris, pH 7.5], followed by overnight incubation at 4 °C with a primary antibody (Table 1) diluted in the blocking buffer, and washed three times with TBS-T [20 mM Tris, pH 7.5, 0.05% (v/v) Tween-20, 150 mM NaCl]. The membrane was incubated for 1 hour at room temperature in the presence of an HRP-conjugated secondary antibody diluted in the blocking buffer, and after washing three times, the enhanced chemiluminescence substrate (ECL) (ClearBand) solution was added. Detection was performed using FUSION SOLO X.

### Immunocytochemistry

Following transfection in the S-GFP construct, SH-SY5Y cells were treated with 200 µl of the exportin 1 inhibitor Leptomycin B (LMB; Sigma, L2913-5UG) at a concentration of 10 ng/ml for 10 minutes. Cells were then fixed with 4% paraformaldehyde (PFA; Sigma, 441244) in PBS for 10 minutes and subsequently rinsed with PBS. In other in vitro experiments, following transfection, cells were washed three times with 1× PBS to remove residual media, immediately fixed with 4% PFA in PBS for 10 minutes, and rinsed with PBS.

Cells were then permeabilized with 0.1% Triton X-100 in PBS for 1 minute and blocked with 5% powdered milk in PBS for 1 hour. This was followed by incubation with the primary antibody (Table 1) for 1 hour, three subsequent rinses, and further incubation with the secondary antibody for 1 hour, followed by three subsequent rinses and 15 min incubation with DAPI. Following rinses, the coverslips were carefully dried and mounted on slides using Immumount (Immu-mount™, Thermo CAT# 9990402). Following overnight drying, samples were ready to be analyzed.

Immortalized fibroblasts derived from SOD1-ALS patients and healthy controls were onto glass-bottom 96-well plates and cultured to ~80% confluence. Cells were fixed with 4% PFA in PBS for 10 minutes at room temperature, rinsed with PBS, stained with primary antibodies diluted in staining solution (0.1% Triton X-100, 1% BSA in 1X HBSS) for 1 hour at room temperature. After three PBS rinses, secondary antibodies were applied for 30 min in staining solution, followed by three additional rinses and DAPI staining for 15 minutes. Cells were stored in PBS for imaging.

### Fluorescent microscopy

Fluorescence measurements were performed on a Nikon TiE inverted microscope driven by the NIS elements software package (Nikon). The microscope was equipped with a Cool Snap HQ2 14bit CCD camera (Roper Scientific), a 40X 0.75 NA Super Fluor objective, a 60X 1.4 NA oil-immersion apochromatic objective (Nikon), a perfect-focus mechanism (Nikon), and EGFP, EYFP, and Cy3 TE-series optical filter sets (Chroma) as well as BFP and Cy5 filter sets (Semrock).

### Human postmortem tissues

Human tissues were obtained using a short-postmortem interval acquisition protocol that followed HIPAA-compliant informed consent procedures and were approved by Institutional Review Board (Benaroya Research Institute, Seattle, WA IRB# 10058 and University of California San Diego, San Diego, CA IRB# 120056). The ALS nervous systems were from patients who presented with ALS as the clinical phenotype and died from respiratory failure. For this investigation, we evaluated 6 control and 4 SOD1 cases.

### Immunofluorescence (IF) in postmortem tissues

Sections were deparaffinized using Citrisolv (Fisher #04–355-121) and rehydrated through a graded ethanol series (1009070%, and 50%). Antigen retrieval was performed in a high-pH buffer (Vector #H-3301) using a pressure cooker at 120 °C for 20 minutes. After cooling on ice for 30 minutes, sections were washed in phosphate-buffered saline (PBSx 1, 3 × 10 minutes), permeabilized, and blocked in a solution containing 5% horse serum and 2% Triton-X (Sigma #65H2616) in PBS for 1 hour at room temperature. Sections were then incubated overnight at 4 °C with primary antibodies (Table 1). After a 30-minute equilibration at room temperature, sections were washed (PBSx1, 3 × 10 minutes) and incubated for 2 hours at room temperature with species-specific Alexa Fluor-conjugated secondary antibodies protected from light. Nuclei were counterstained with DAPI for 5 minutes, and autofluorescence was quenched with TrueBlack in 70% ethanol (1 × 15 seconds). Slides were mounted with ProLong Gold antifade mounting medium containing DAPI and imaged within 24–48 hours using an Olympus VS200 microscope at 20× magnification.

### Image acquisition and analyses of human post mortem tissues

For RanGAP1, Slides were scanned with Hamamatsu Nanozoomer 2.0HT Slide Scanner at 40X magnification. Additionally, neurons were visualized using the fast mode for Zeiss 800 laser scanning microscope with airyscan, under 40X water magnification. Neurons were quantified only if the nucleus of a neuron was present in the imaged plane. Maximum projections of z-stacks were compiled using Zen Black. The analysis included neurons per case ranging from 2 to 37. RanGap1 intensity was automatically quantified using Image J scripts.

For XPO1 and FG-Nups, slides were carefully cleaned and loaded onto the scanner trays, ensuring proper orientation and alignment. Fluorescence Expert Scan mode was selected to allow for editable scan parameters. An overview image was obtained using brightfield at 2X magnification to define scan areas and focus points. Each tissue section was enclosed within the scan area, with prefocus points set and non-sample regions included. For high-resolution acquisition, fluorescence channels including DAPI, TRITC and Cy5 were selected, with DAPI configured as the first channel and undersaturated below 65535 for optimal quantification. Manual exposure settings were used, and exposure times were adjusted while viewing live camera images, ensuring they remained below 500 ms to maintain scan efficiency. Final scans were performed at 20X magnification under normal Z-plane settings. Processed images were reviewed using the Image Processing tab, with intensity adjustments applied if necessary. High-resolution images were saved to the local drive in the appropriate format and renamed in accordance with protocol guidelines to avoid errors in later access. For image analysis and quantification, motor neurons of the anterior horn were manually segmented. Motor neurons of the anterior horn were manually segmented. For NPC intensity analysis, the nucleus of each neuron was identified and the intensity of a 1-pixel wide area (approx. 0.3 μm) around the nuclear envelope was measured. Measurements of each cell were normalized by dividing nuclear envelope measurement by the background value as measured by the nucleoplasm intensity. XPO-1 intensity was measured in the same neurons. In addition to nuclear XPO-1 signal, cytoplasmic XPO-1 was measured in the 1.5-pixel wide region (approx. 4.5 μm) immediately surrounding the nucleus. Microglia were identified by positive Iba1staining. Their nucleus was segmented and XPO-1 intensity from the nucleus and the remaining cytoplasmic region were measured to determine the C/N ratio. All segmentation and measurement were done with Fiji [107].

### Immunofluorescence staining for imaging flow cytometry

Fibroblasts were trypsinized, collected, and centrifuged at 800 g for 5 min. The pellet was washed with 1X PBS and strained through a 70-µm nylon mesh strainer (Corning). Cells in suspension were fixed with 2% paraformaldehyde in 1X PBS-EDTA 0.02% for 20 min at room temperature. After centrifugation and two 1X PBS washes, cells were permeabilized in 0.2% Triton-X-100 for 15 min and blocked with Human BD Fc block (BD Bioscience) for 10 min. Staining with RanGAP1 or Lamin B1 primary antibody (Table 1) occurred for 1 hour, followed by a 30-minute incubation with the secondary antibody. The cells were incubated with DAPI (Thermo Fisher) for 10 min, centrifuged, and washed with 1X PBS, then resuspended in 1X PBS-EDTA 0.02%. Prior to imaging flow cytometry, cells were filtered with a 70-µm cell strainer and collected into a 1.5-ml siliconized polypropylene microcentrifuge tube pre-washed with 1% BSA.

Cells stained with RanGAP1 or Lamin B1 antibody were imaged using the Amnis ImageStream®X Mk II (ISX) imaging flow cytometer (Amnis, Luminex Corporation). Data acquisition was performed at 40X magnification. Prior to the experiments, the cytometer underwent calibration, and confirmation of its compliance with all internal quality control tests was done. Subsequently, cells were gated based on the best focus in the bright-field (BF) channel and further refined to single cells using the BF of the focused population.

The acquired raw image files (.rif) were imported into the IDEAS® package associated with the ISX for the generation of a compensated image file (.cif) and a data analysis file (.daf). Focused cells were gated using the gradient RMS feature to ensure the selection of high-quality images. Single cells were gated by excluding debris, doublets, and aggregates. Quantification of cytoplasmic RanGAP1 or Lamin B1 involved measuring the median intensity of a tight nuclear mask for DAPI-stained nuclei, subtracted from the whole cell mask using Boolean Logic (“Whole Cell And Not Nucleus”). Cell circularity was estimated by the circularity of the mask Erode, utilizing Lamin B1 staining.

### SOD1 transgenic mice

In this study, we used transgenic mice expressing the human SOD1^G93A^, SOD1^G85R,^ and loxSOD1^G37R^ that were previously described [10, 108, 109]. All mouse lines were bred on a pure C57BL6 background to eliminate confounding genetic influences. Mice were genotyped by PCR using DNA extracted from a tail biopsy. All mice were maintained, employing standard protocols, in the Ben-Gurion University of the Negev animal facility. All procedures involving animals were consistent with the requirements of the Animal Care and Use Committees of the Ben-Gurion University of the Negev.

The SOD1^G93A^ mouse model in the pure C57BL/6 background used here, carries approximately 20–25 copies of the human SOD1 gene with the G93A mutation, driven by its endogenous promoter. This model is known to exhibit elevated SOD1 protein expression (5- to 15-fold higher than endogenous levels), resulting in progressive motor neuron degeneration, neuromuscular junction denervation, and gliosis. Onset of motor symptoms typically occurs at ~92–110 days, with disease progression leading to end-stage paralysis by ~150–160 days [110]. LoxSOD1^G37R^ transgenic mice (C57BL/6 background) carrying a single-copy human SOD1 gene with the G37R mutation, expressed under the control of its endogenous promoter, were also used. These mice express the mutant protein at levels only modestly above endogenous SOD1 and develop a more slowly progressing disease. Disease onset occurs at approximately 238–241 days, with end-stage paralysis at ~397–408 days [110, 111]. In addition to the SOD1^G93A^ and SOD1^G37R^ models, the SOD1^G85R^ transgenic mouse model was also used to investigate ALS-related pathology. This model expresses a low-copy (1–2 copies) transgene encoding the human SOD1 gene with the G85R mutation, under the control of its endogenous promoter, on a C57BL/6 background. Unlike high-copy models, SOD1^G85R^ mice exhibit mutant SOD1 expression levels that are comparable to or slightly elevated above endogenous levels. This model is particularly notable for producing misfolded and aggregation-prone SOD1 protein with no dismutase activity, making it a widely used tool for studying SOD1 proteinopathy and aggregation dynamics in ALS. Clinically, SOD1^G85R^ mice show a relatively late disease onset (~250–300 days), followed by a progressive rapid motor decline leading to paralysis and end-stage at approximately 350–400 days [111, 112].

### Spinal cord tissues from subpial injected mice

Fixated spinal cord tissues from 3 groups of mice of Bravo-Hernandez et al. [64] were used: (1) SOD1^G37R^ sham-operated mice (*n* = 3; age ~396 d); (2) SOD1^G37R^ mice subpially treated in their presymptomatic stage (~120d) with AAV9–shRNA–SOD1 (*n* = 3, age ~472 d); (3) wild-type non-transgenic mice (*n* = 4; age~ 480). The sham group consisted of animals that underwent surgical manipulation without AAV injection or CNS transduction. As such, we cannot fully rule out the possibility that the observed changes in our experiments may be partially influenced by AAV transduction.

### Perfusion and tissue processing

Mice were anesthetized via inhalation of 1.5–3% Isoflurane, followed by 50-75 mL 0.1 M PBS transcardiac perfusion, then switched to 4% paraformaldehyde in PBS. The spinal cords were dissected out and post-fixed in 4% formaldehyde at 4 °C overnight, incubated in 20% sucrose for 48 h at 4 °C and cryo-embedding was performed in Optimal Cutting Temperature (OCT) matrix compound (Tissue-Tek, Sakura Finetek). Tissues were then cryo-sectioned to free-floating sections 30 µm thick and stored at 4 °C PBSX1 with sodium azide 0.05%.

### Immunohistochemistry (immunofluorescence)

Sections were rinsed twice in PBS with 0.3% triton for permeabilization and then in PBS. Next, sections were blocked for 1 h in blocking solution (1X PBS, 1% free fatty acid BSA, 0.3% Triton-X100, and 10% donkey serum (Abcam), immunostained with primary antibodies (Table 1) diluted in 1X PBS, with 0.3% Triton-X100 and 5% donkey serum, and incubated overnight at 4 °C with gentle agitation. The following day, sections were washed three times in PBS and incubated for 1.5 h at room temperature with fluorescent conjugated secondary antibodies (Table 1) diluted in 1X PBS, with 0.3% Triton-X100 and 5% serum. Lastly, DAPI (Sigma) diluted in PBS was added for nuclear staining. Sections were washed (3 × 15 min) in PBS and mounted on slides using Immu-Mount^TM^ mounting solution (Thermo), dried at room temperature overnight, and stored at 4 °C until imaging.

### Confocal microscopy

Images were acquired on a NIKON C2Plus laser unit dock to a Nikon Eclipse *Ti* unit of the confocal microscope by using a 20X and 60X oil immersion objective for the mouse tissues, and 40X oil immersion objective for the fibroblasts. Scanning settings were reused across the samples. Fluorescence intensity was quantified using NIS Element AR 5.21.03 software.

### Image processing and quantification

To measure fluorescent intensities, the mean intensity of the ROI was taken, and the background signal was subtracted. DAPI staining was used as a reference for determining nuclear boundaries, and the cytosolic boundaries were determined by the ChAT/NeuN staining. To measure the nuclear envelope intensity of RanGAP1 and FG-Nups, we used the DAPI staining to define the inner ring, and the dilate operation of this ring is used to define an outer ring boundary (1.3 $\mu$ m dilation). The measured intensity of the nuclear envelope corresponded to the area between the two rings.

For fibroblasts, images were analyzed using CellProfiler (version 4.2.6). Nuclear and cytoplasmic compartments were segmented based on DAPI and Vimentin. Per-well median intensity values were extracted from individual cells for each marker, including TDP-43, XPO1, misfolded SOD1, Ran, RanGAP1, and FG-Nups. Custom pipelines were developed for automated object identification and measurement of per-cell intensities. Results were exported for further analysis and normalization. RanGAP1 cytoplasmic puncta in fibroblasts were quantified using CellProfiler by applying intensity thresholds and size constraints to exclude background or noise artifacts. All thresholding and segment parameters were held constant within each experiment.

FG-Nups puncta in tissue samples were considered if the signal was not uniformly distributed around the nucleus and was accumulated in a round shape close to the nuclear envelope. The nuclear circularity was defined using FG-Nups and Lamin B staining and analyzed by ImageJ program. Colocalization between Iba1/CD68 and XPO1 was measured by segmentation with the thresholding of the two separate channels, followed by examination of the overlapping area in each image. Colocalization ratio was determined as the ratio between the size of the overlapping area and the size of the Iba1/CD68 area per image. Fluorescence intensity values were normalized to the means of the control condition in each independent experiment. Quantifications were performed using NIS Element AR 5.21.03, ImageJ, and Cell Profiler, depending on the dataset and experimental context.

### Statistical analysis

Statistical parameters and tests used are noted in figure legends. All experiments included at least three biological repeats. Images and micrographs are representative of all experimental repeats. Our statistical analysis was designed to simultaneously address three primary challenges: non-normality distribution in some data, family-wise error in multiple comparisons, and potential within-cluster correlations. To account for non-normality, we implemented a rank-based non-parametric methodology. For comparisons involving more than two groups, we conducted a one-way analysis of variance (ANOVA) on ranks. In cases where precisely two groups were compared, we employed two-sample t-tests. To mitigate family-wise error rate in multiple comparisons, we applied Tukey’s post-hoc method. This approach enabled us to identify specific group differences while maintaining control over Type I errors. To adjust for the clustered nature of our data, we calculated p-values using robust standard errors. This method accounts for potential correlations within clusters, ensuring the validity of our statistical inferences. All statistical analyses were performed using R version 4.3.3 software. Significance was set at a confidence level of 0.05. In all figures, non-significant denotes *p*≥0.05, * denotes *p* < 0.05, ** *p* < 0.01, *** *p* < 0.001.

**Table 1:** Antibodies used for immunoblot and immunofluorescence analyses

IDENTIFIER	SOURCE	dilution	Antibodies
AB144P	Merck Millipore	1:100	Choline Acetyltransferase (ChAT), goat
GTX113164	Genetex	1:500	Choline Acetyltransferase (ChAT), rabbit
MM-0070	Medi Mabs	1:100	B8H10 (misfolded SOD1), mouse
SC-28322	Santa-Cruz	1:100	RanGAP1 (C-5), mouse
SC-271376	Santa-Cruz	1:100	Ran-GTP (a-7), mouse
BLG-902901	Biolegend	1:200	FG-Nups (mAB414), mouse
SC-74454	Santa-Cruz	1:100	XPO1 (C-1), mouse
A300-469A	Bethyl	1:100	XPO1, rabbit
10782–2-AP	Proteintech	1:200	TDP-43, rabbit
ARP-38942	Aviva Systems Biology	1:500	TDP-43, rabbit (for Fibroblasts)
Vim	AvesLabs	1:500	Vimentin, chicken
AB5076	Abcam	1:400	Ionized calcium-binding adaptor molecule 1 (Iba1), goat
ABR-PA316727	Thermo	1:100	GFAP, rabbit
MABN140	Merck Millipore	1:200	Neuronal nuclei antigen (Neun)
SC28322	Santa-Cruz	1:100 (in fibroblast)/1:250 (in postmortem tissue)	RanGAP1, Mouse
ab16048	Abcam	1:500	Lamin B1, Rabbit
sc-101523	Santa-Cruz	1:500	SOD1(24), Mouse
ab6046	Abcam	1:400	β- tubulin, Rabbit
ab178846	Abcam	1:400	Iba1
46249	Cell Signaling Technology	1:200	XPO1, Rabbit
A31572	Thermo	1:350	donkey anti-rabbit, Alexa fluor 555
A31570	Thermo	1:350	donkey anti-mouse, Alexa fluor 555
A21443	Thermo	1:350	Chicken anti-Rabbit, Alexa Fluor 647
AB-ab150111	Abcam	1:200	Donkey anti-mouse, Alexa fluor 647
AB-ab150135	Abcam	1:200	Donkey anti-goat, Alexa fluor 647
715–545-150	Jackson Immuno Research	1:500	donkey anti-mouse, Alexa 488
A-21207	Invitrogen	1:500	donkey anti-rabbit, Alexa 594
705–605-147	Jackson Immuno Research	1:500	donkey anti-goat, Alexa 647
A21103	Invitrogen	1:1000	Goat anti-chicken, Alexa 633
A10037	Thermo	1:300	Donkey anti-mouse Alexa Fluor 568
A32790	Thermo	1:300	Donkey anti rabbit Alexa Fluor 488
A21447	Thermo	1:300	Donkey anti goat Alexa Fluor 647
115–035-166	Jackson	1:5000	Goat anti-mouse HRP
111–035-144	Jackson	1:5000	Goat anti-rabbit HRP

## Electronic supplementary material

Below is the link to the electronic supplementary material.

Supplementary Material 1

Supplementary Material 2

Supplementary Material 3

## Data Availability

All data generated or analyzed during this study are included in this published article and Supplementary Figures

## Abbreviations

NCT: Nucleocytoplasmic transport
ALS: Amyotrophic lateral sclerosis
SOD1: Superoxide dismutase 1
SALS: Sporadic ALS
FALS: Familial ALS
NPC: Nuclear pore complex
Nups: Nucleoporins
FG-Nups: Phenylalanine-glycine repeat domain nucleoporins
NLS: Nuclear localization sequence
NES: Nuclear export sequence
FTD: Frontotemporal dementia
C9orf72: Chromosome 9 open reading frame 72
DPR: Dipeptide repeats
TDP-43: TAR DNA-binding protein 43
FUS: Fused in sarcoma
PFN1: Profilin 1
S-GFP: Shuttle-GFP (NLS-NES-GFP reporter)
XPO1: Exportin 1
LMB: Leptomycin B
Iba-1: Ionized calcium-binding adaptor molecule 1
GFAP: Glial fibrillary acidic protein
ROS: Reactive oxygen species
ChAT: Choline acetyltransferase
AAV: Adeno-associated virus
shRNA: Short hairpin RNA
ER: Endoplasmic reticulum
