# Supplementary material for: Impaired nucleocytoplasmic transport in SOD1-mediated ALS
Source: Mol Neurodegener. 2026 Feb 14;21:14. doi: 10.1186/s13024-026-00930-8 (PMC12922372; doi:10.1186/s13024-026-00930-8)
Supplement: Supplementary file 1 — Supplementary Material 1 [file 13024_2026_930_MOESM1_ESM.docx]

**Supplementary figure legends**

**Supplementary Fig. 1. Misfolded SOD1 accumulates in the cytosol of SH-SY5Y** **cells and motor neurons expressing mutant SOD1. a.** Confocal imaging of SH-SY5Y cells expressing SOD1^WT^ or SOD1^G93A^, stained with antibody against misfolded SOD1 (magenta; B8H10) and DAPI (blue). Scale bar, 20 µm**. b.** Quantification of misfolded SOD1 intensity in the cytosol and the nucleus from (**a**). **c.** Zoomed-out view of the images shown in Figure 1c. The insets represented in Figure 1c are indicated by white dashed-line squares. Scale bar, 30 μm **d.** Immunofluorescence of lumbar spinal cord sections from symptomatic SOD1^G93A^ and non-transgenic mice stained with another antibody against misfolded SOD1 (magenta; SE-21), ChAT (green) and DAPI (blue). Scale bar, 25 µm.  **e.** Quantification of misfolded SOD1 in the cytosol and nucleus of spinal motor neurons from symptomatic SOD1^G93A^ mice (n=3). Graphs represent quartiles (box), 50th percentiles (center lines) and range (10-90; whiskers). Three independent experiments for (**b**) (dots represent cells from all experiments. Rank-based Two-samples t-test, p-values adjusted for clustering, ***P < 0.001), three independent experiments for (**e**) (dots represent motor neurons from all experiments. Rank-based Two-samples t-test, p-values adjusted for clustering; ***P < 0.001).

**Supplementary Fig. 2. XPO1 distribution is altered in the spinal cord of mutant SOD1^G85R^ mice. a.** Immunofluorescence of lumbar spinal cord sections from non-transgenic and late symptomatic SOD1^G85R^ mice stained for anti-XPO1 (magenta), anti-ChAT (green), and DAPI (blue). Scale bar, 10 µm. **b.** Quantification of cytosol-to-nucleus ratio of XPO1 distribution from data in (**a**). **c.** Quantification of XPO1 nuclear levels represented in (**a**). Graphs represent quartiles (boxes) with data overlap, 50th percentiles (center lines) and range (10-90; whiskers). Three independent experiments (n=3) for (**b**) and (**c**) (dots represent quantified motor neurons from all experiments: ***P < 0.0001, Mann-Whitney test).

**Supplementary Fig. 3. Cytoplasmic accumulation of misfolded SOD1 in motor neurons is associated with XPO1 abnormalities in the spinal cord of mutant SOD1^G93A^ mice. a, c.** Immunofluorescence of lumbar spinal cord sections from non-transgenic (n=4) and symptomatic mutant SOD1^G93A^ (n=4) mice, stained with anti-XPO1 (**a**) or B8H10 anti-misfolded SOD1 (**c**) antibodies (magenta), anti-ChAT (green), and DAPI (blue). Adjacent sections were obtained from the same spinal cords for the non-transgenic and for the SOD1^G93A^ mice. Scale bar, 20 µm. **b.** Quantification of nuclear XPO1 intensity in spinal motor neurons from data in (**a**). **d.** Quantification of cytosolic misfolded SOD1 intensity in spinal motor neurons from data in (**c**). **e.** Confocal imaging of SH-SY5Y cells expressing SOD1^WT^ or SOD1^G93A^ stained with an antibody against XPO1 (magenta) and DAPI (blue). Scale bar, 20 µm. **f.** Quantification of nuclear intensity of XPO1 presented in (**e**). **g.** Immunoblot analysis from cells described in (**e**). Top panel shows SOD1 overexpression in cells transfected with SOD1^WT^ and SOD1^G93A^ constructs compared to control. Bottom panel shows β-tubulin as a loading control. Graphs represent quartiles (boxes) with data overlap, 50th percentiles (center lines) and range (10-90; whiskers). Three independent experiments (n=3) for (**b**) and (**d**) (dots represent quantified motor neurons from all experiments: ***P < 0.0001, Kruskal Wallis ANOVA). Three independent experiments for (**f**) (dots represent quantified cells from all experiments; n.s., non-significant. Mann-Whitney test).

**Supplementary Fig. 4. L38R mutation in SOD1^G93A^ restores nuclear export function. a.** SOD1^G93A^ sequence, before (top) and after (bottom) insertion of a mutation in the NES-like sequence. The NES-like sequence is highlighted in a green rectangle, including the residues 35-47. Amino acid mutations L38R and G93A are represented in magenta. Black bold residues in the NES-like sequence are five hydrophobic residues essential for protein export. **b.** 3D structure of SOD1^G93A/L38R^. Mutations are highlighted in magenta, and the residues of the NES-like sequence are labeled in green with the five hydrophobic residues in black. This figure was prepared with UCSF Chimera and PDBePISA, based on SOD1 crystal structures available in RCSB Protein Data Bank (PDB ID: 3GZO, HUMAN SOD1 G93A Variant, only chain A of the dimer was shown). **c.** SH-SY5Y cells expressing S-GFP (green) and SOD1^WT^ (control), SOD1^G93A^_,_ or SOD1^G93A/L38R^. Accumulation of misfolded SOD1 was detected by B8H10 antibody (magenta); DAPI (blue). **d.** Quantification of S-GFP cytosol-to-nucleus ratio from data represented in (**c**). Graphs represent quartiles (boxes) with data overlap, 50th percentiles (center lines) and range (10-90; whiskers). Three independent experiments (n=3) for (**d**) (dots represent raw data from all experiments: ***P < 0.0001, Kruskal- Wallis ANOVA).

**Supplementary Fig. 5. Exportin 1 accumulates in the cytoplasm of cells surrounding the motor neurons in the spinal cord of symptomatic SOD1^G93A^ mice. a.** Immunofluorescence of lumbar spinal cord sections from symptomatic SOD1^G93A^ mice stained with anti-XPO1 (magenta), anti-ChAT (green), and DAPI (blue). Scale bar, 20 µm. Arrows represent the observed DAPI staining surrounded by XPO1. **b, c.** Intensity profile plots. An intensity profile plot line (white dashed arrow) was drawn through Iba1-positive cell from the spinal cord of presymptomatic SOD1^G93A^ mice, and signal intensity was plotted across the length of the line to determine the co-localization of XPO1 and Iba1 proteins within microglia. Scale bar, 5 µm. **d, e.** Intensity profile plots. An intensity profile plot line (white dashed arrow) was drawn through Iba1-positive cell from the spinal cord of end-stage SOD1^G93A^ mice, and signal intensity was plotted across the length of the line to determine the co-localization of XPO1 and Iba1 proteins within microglia. Scale bar, 5 µm.

**Supplementary Fig. 6. Exportin 1 accumulates in the cytoplasm of microglia in the spinal cord of SOD1^G37R^ and SOD1^G85R^ mice. a, b.** Colocalization analysis using immunofluorescent staining of lumbar spinal cord sections from non-transgenic, symptomatic SOD1^G37R^ (**a**) and symptomatic SOD1^G85R^ (**b**) transgenic mice, stained with anti-XPO1 (magenta), anti-Iba1 (green), and DAPI (blue). Scale bar, 20 µm. **c, d.** Quantification of Iba1 and XPO1 overlap staining in (**a**) and (**b**), respectively. **e.** Quantification of the number of microglia as detected by Iba1 staining from ten images of each spinal cord sample (n=3 per group) during disease progression. Graphs represent quartiles (boxes) with data overlap, 50th percentiles (center lines) and range (10-90; whiskers). Three independent experiments (n=3) for (**c**) and (**d**) (each dot represents an image; n.s. non-significant, **P < 0.01, Two-sample t-test). Three independent experiments (n=3) for (**e**) (dots represent each spinal cord sample, n.s. non-significant, **P < 0.01, ***P<0.001, One-Way ANOVA Test).

**Supplementary Fig. 7. Exportin 1 accumulates in the cytoplasm of activated microglia in the lumbar spinal cord of mutant SOD1^G93A^ mice. a.** Colocalization analysis using immunofluorescent staining of lumbar spinal cord sections from end-stage SOD1^G93A^ mice with antibodies against XPO1 (magenta), CD68 (green), and DAPI (blue). Scale bar, 20 µm. **b, c.** Intensity profile plots. An intensity profile plot line (white dashed arrow) was drawn through CD68-positive cell from the spinal cord of end-stage SOD1^G93A^ mice, and signal intensity was plotted across the length of the line. Scale bar, 5 µm. **d.** Quantification of CD68 and XPO1 overlap in (**a**). **e.** XPO1 RNA expression in microglia isolated from spinal cord of SOD1^WT^ and three spinal cords of SOD1^G93A^ mice [65]. In this study a more aggressive version of the SOD1-G93A model was used, bred on a B6SJL background, with an earlier onset (~90 days) and shorter lifespan (~130 days). **f.** BacTRAP analysis of ribosome affinity–purified spinal motor neurons [66] from non-transgenic (Control) and SOD1^G37R^ mice (LoxSOD1^G37R^, carrying a single-copy human SOD1 gene with the G37R mutation, with disease onset at ~238–241 days, and end-stage paralysis at ~397–408 days). **g.** Quantification of XPO1 intensity ratio between the cytosol and the nucleus of Iba1 positive cells from Figure 3a. **h.** Quantification of the total XPO1 intensity in Iba1 positive cells showing increased amount as disease progresses as shown in Figure 3a. **i**. Zoomed-out view of the images shown in Figure 4c. The insets represented in Figure 4c are indicated by white dashed-line squares. Scale bar, 30 μm. Graphs represent quartiles (boxes) with data overlap, 50th percentiles (center lines) and range (10-90; whiskers). Four independent experiments **(**n=4**)** for (**d**) (each dot represents a colocalization image from raw data; n.s. non-significant, Two-sample t-test). Bars represent SEM. In (**e**) and (**f**) (dots represents expression value for each sample: n.s, non-significant, *P<0.05). For (**g**) and (**h**) dots represent quantified XPO1 intensity of Iba1-positive microglia cells from all the experiments. (Kruskal Wallis ANOVA; *P < 0.05, **P<0.01, ***P < 0.001).

**Supplementary Fig. 8. SOD1 pathology correlates with impaired nuclear import and loss of nuclear pore components.**  **a.** Confocal imaging of SH-SY5Y cells expressing SOD1^WT^ or SOD1^G93A^ stained with antibody against RanGAP1 (magenta) and DAPI (blue). Scale bar, 20 µm. **b.** Quantification of laminar RanGAP1 intensity from (a). c, d. Zoomed-out view of the images shown in Figures 5a and 5d. The insets represented in Figures 5a and 5d are indicated by white dashed-line squares. Scale bar, 30 μm. Graphs represent quartiles (boxes) with data overlap, 50th percentiles (center lines) and range (10-90; whiskers). Three independent experiments for (**b**) (dots represent cells from all experiments, ***P<0.001, Mann-Whitney).

**Supplementary Fig. 9. RanGAP1, but not TDP-43, accumulates in the cytosol of microglia in the lumbar spinal cord of mutant SOD1 mice. a.** Immunofluorescent staining of lumbar spinal cord sections from non-transgenic (n=3) and end-stage SOD1^G93A^ (n=3) mice, with antibodies against RanGAP1 (magenta), Iba1 (green), and DAPI (blue). Scale bar, 10 µm. **b.** Quantification of Iba1 and RanGAP1 staining overlap in (**a**). **c, d.** An intensity profile plot line (white dashed arrow) was drawn through Iba1-positive cells (**c**), and signal intensity was plotted across the length of the line to determine the co-localization of RanGAP1 and Iba1 proteins within microglia (**d**). Scale bar, 5 µm. **e.** Immunofluorescent staining of lumbar spinal cord sections from non-transgenic (n=3) and end-stage SOD1^G93A^ (n=3) mice, with antibodies against TDP-43 (magenta), Iba1 (green) and DAPI (blue). Scale bar, 25 µm. **f.** Quantification of Iba1 and TDP-43 staining overlap in (**e**). **g, h.** An intensity profile plot line (white dashed arrow) was drawn through Iba1-positive cells (**g**), and signal intensity was plotted across the length of the line to determine the co-localization of TDP-43 and Iba1 proteins within microglia (**h**). Scale bar, 5 µm. Graphs represent quartiles (boxes), 50th percentiles (center lines) and range (10-90; whiskers). Three independent experiments for (**b**) and (**f**) (each dot represents a colocalization image; *P<0.05, ***P<0.001, Mann-Whitney test).

**Supplementary Fig. 10. Lack of misfolded SOD1 detection in Iba1-positive microglia *in vivo* and *in vitro*.** **a.** Immunofluorescent staining of lumbar spinal cord sections from mutant SOD1^G93A^ mice (n=3) at the symptomatic stages of the disease, with antibodies against B8H10 (magenta), Iba1 (green), and DAPI (blue). Scale bar, 30 µm. **b, c.** An intensity profile plot line (white dashed arrow) was drawn through Iba1-positive cells (**b**), and signal intensity was plotted across the length of the line to determine the co-localization of B8H10 and Iba1 proteins within microglia (**c**). Scale bar, 10 µm. **d.** Immunostaining of primary microglia stained with antibodies against Iba1 (green), misfolded SOD1 (magenta; B8H10) and DAPI (blue).

**Supplementary Fig. 11. FG-Nups intensity is reduced in mutant SOD1-expressing SH-SY5Y cells.** **a.** Zoomed-out view of the images shown in Figure 5f. The insets represented in Figure 5f are indicated by white dashed-line squares. Scale bar, 30 μm. **b.** Confocal imaging of SH-SY5Y cells expressing SOD1^WT^ or SOD1^G93A^, stained with antibody against FG-Nups (magenta; Mab414) and DAPI (blue). Scale bar, 20 µm. **c.** Quantification of laminar intensity of FG-Nups from (**b**). Graphs represent quartiles (box), 50th percentiles (center lines) and range (10-90; whiskers). Three independent experiments (dots represent cells from all experiments). Rank-based Two-samples t-test, p-values adjusted for clustering, ***P < 0.001).

**Supplementary Fig. 12.** **Mutant SOD1 does not alter nuclear morphology but disrupt the nuclear membrane integrity in the spinal cord of SOD1^G93A^ mice. a.** Immunofluorescence of endogenous ring-shaped FG-Nups (mAb414) (magenta) in ChAT-positive cells (green) from lumbar spinal cord sections of non-transgenic (n=3) and end-stage SOD1^G93A^ (n=3) mice. DAPI (blue) was used to detect the nucleus. Scale bar, 10 µm. **b.** Circularity analysis of the laminar intensity of FG-Nups in (**a**). **c.** An illustration of the fragmentation analysis used to quantify the continuity of FG-Nups staining around the perimeter of the nucleus. **d.** Quantification of continuous versus discontinuous (fragmented) FG-Nups signals in the nuclei of motor neurons in (**a**). **e.** Population pyramid plot showing the percentage distribution of motor neuron nuclei across different numbers of FG-Nups signal fragments per neuron. Each bar represents the average percentage across all cells pooled from all experiments. **f.** Quantification of the Continuity Index from discontinuous membranes (as described in panel **c**) based on FG-Nups staining in spinal motor neuron nuclei from control and SOD1^G93A^ mice. Graphs represent quartiles (boxes) with data overlap, 50th percentiles (center lines) and range (10-90; whiskers). Three independent experiments for (**b**) (dots represent raw data from all experiments; n.s. non-significant, Mann-Whitney test. Bars represent mean ± SEM. Three independent experiments for (**d**) (dots represent average number of cells in each spinal cord examined; **P<0.01, One-Way ANOVA). Three independent experiments for (**e**) **(**dots represent average number of discontinuous cell membrane in each spinal cord examined; ***P<0.001 Two-Sample T-test).

**Supplementary Fig. 13.** **No evidence of Ran-GTP, TDP-43, or XPO1 mislocalization in SOD1-ALS patient-derived fibroblasts. a.** Size of RanGAP1 inclusions in fibroblasts from six healthy controls and six SOD1-ALS patients (five SOD1-A4V and one SOD1-D90A) using confocal imaging. The box plot reflects median per well data, and each data point indicates the overall average for each line. Unpaired t-test comparing the overall average of each line from three independent experiments; n.s. non-significant. **b.** Representative confocal images of immortalized fibroblasts from SOD1-ALS patients and controls immunostained for RanGTP (gray) and DAPI (blue). Scale bar, 20 µm. **c.** Quantification of the cytoplasmic-to-nucleus intensity ratio of RanGTP in six SOD1-ALS patients (five SOD1-A4V, one SOD1-D90A) and six healthy controls. **d, f.** Representative confocal images of immortalized fibroblasts immunostained for XPO1 or TDP-43 (gray) and DAPI (blue). Scale bar, 20 µm. **e, g.** Quantification of the cytoplasmic-to-nucleus XPO1 and TDP-43 intensity ratio reveals no significant difference between patient (five SOD1-A4V, one SOD1-D90A) and control lines. Graphs represent quartiles (box), 50th percentiles (center lines) and range (10-90; whiskers). For (**c**), (**e**) and (**g**), the box plot reflects median per well; each data point reflects median per line. Statistical significance was assessed across data points. Unpaired t-test; n.s. non-significant**.**

**Supplementary Fig. 14. No detection of misfolded SOD1 and normal nuclear membrane shape in SOD1-mutant fibroblasts. a.** Immunofluorescence of fibroblasts from two SOD1-ALS patients (SOD1-D90A and SOD1-A4V) and two healthy controls stained for Lamin B1 (magenta) and DAPI (blue). Scale bar, 10 μm. **b.** Nuclear circularity of controls and SOD1-ALS patients determined using Lamin B1 immunostaining in (**a**). The box plot reflects median per cell; each data point reflects median per well. Statistical significance was assessed across each data point. Unpaired t-test; n.s. non-significant **c.** Flow cytometry-based imaging of fibroblasts stained with Lamin B1 (red) and DAPI (blue). Bright field is shown in gray. Scale bar, 10 μm. Flow cytometry-based imaging analysis measuring the circularity (**d**) and the cytosolic intensity of Lamin B1 (**e**) from two healthy controls, one SOD1-A4V, and one SOD1-D90A ALS patient. **f.** Confocal imaging of fibroblast lines immunostained with the B8H10 antibody against misfolded SOD1 (gray) and DAPI (blue) does not detect accumulation of misfolded SOD1 in patient or control cells. Scale bar, 20 µm. Bars represent mean ± SEM. Each dot represents the average from a single experiment (three experiments per line); squares indicate data from the SOD1-D90A line. Statistical analysis was performed using an unpaired t-test comparing the experimental averages for each line (n.s. non-significant, student t-test).

**Supplementary Fig. 15. Increased laminar RanGAP1 accumulation in spinal motor neurons of SOD1^A4V^ ALS patients. a.** Immunofluorescent staining of RanGAP1 imaged by NanoZoomer microscope. Scale bar, 20 μm **b.** Quantification of laminar RanGAP1 intensity from (**a**). Graph represents quartiles (box) with data overlap, 50th percentiles (center lines) and range (10-90; whiskers). Data was analyzed from four healthy controls and four SOD1^A4V^ ALS patients (dots represent raw data from all experiments; ***P < 0.001, Mann-Whitney test).
