# Supplementary material for: Impaired nucleocytoplasmic transport in SOD1-mediated ALS
Source: Mol Neurodegener. 2026 Feb 14;21:14. doi: 10.1186/s13024-026-00930-8 (PMC12922372; doi:10.1186/s13024-026-00930-8)
Supplement: Supplementary file 2 — Supplementary Material 2 [file 13024_2026_930_MOESM2_ESM.pdf]

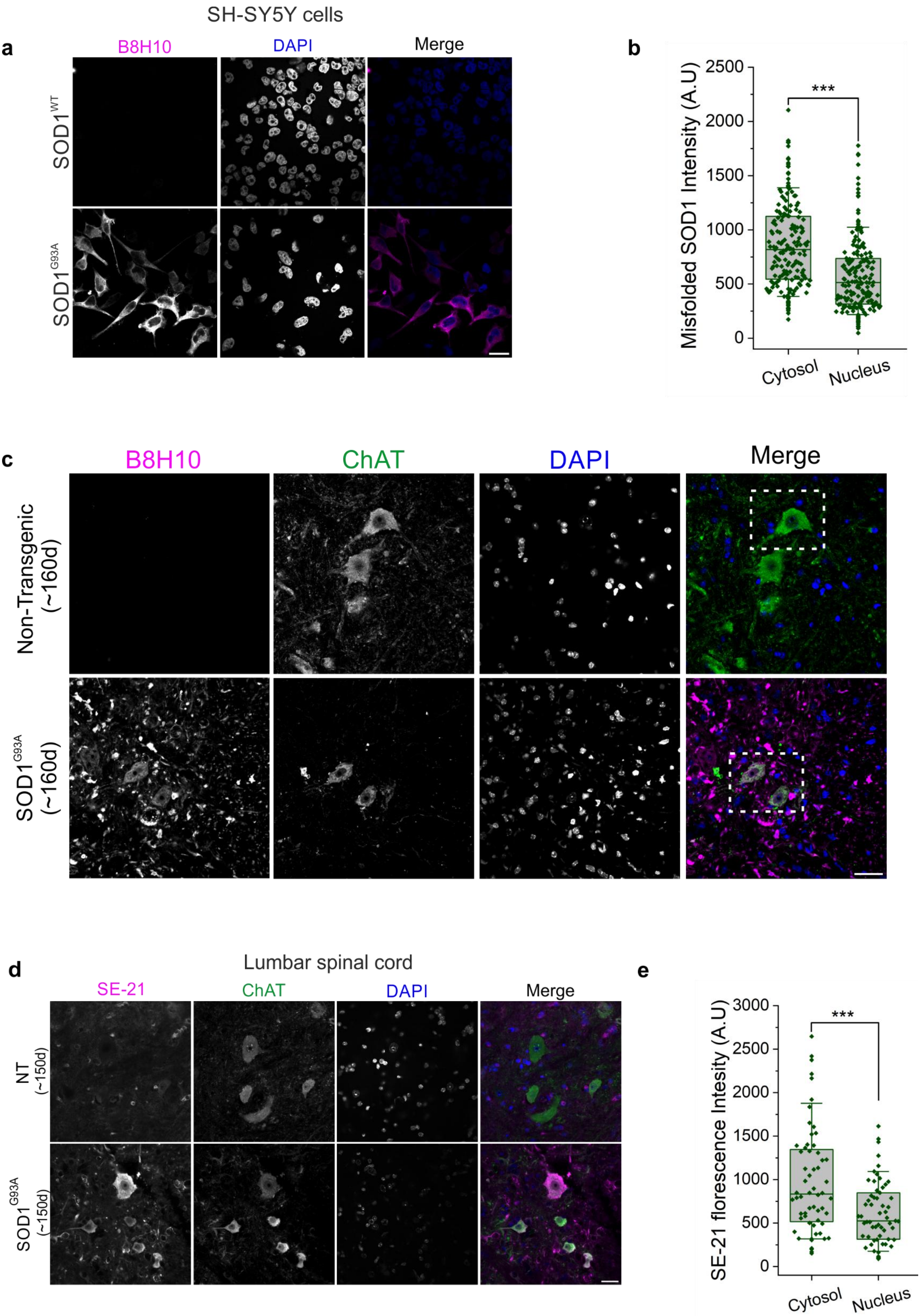

Supplementary Figure 1

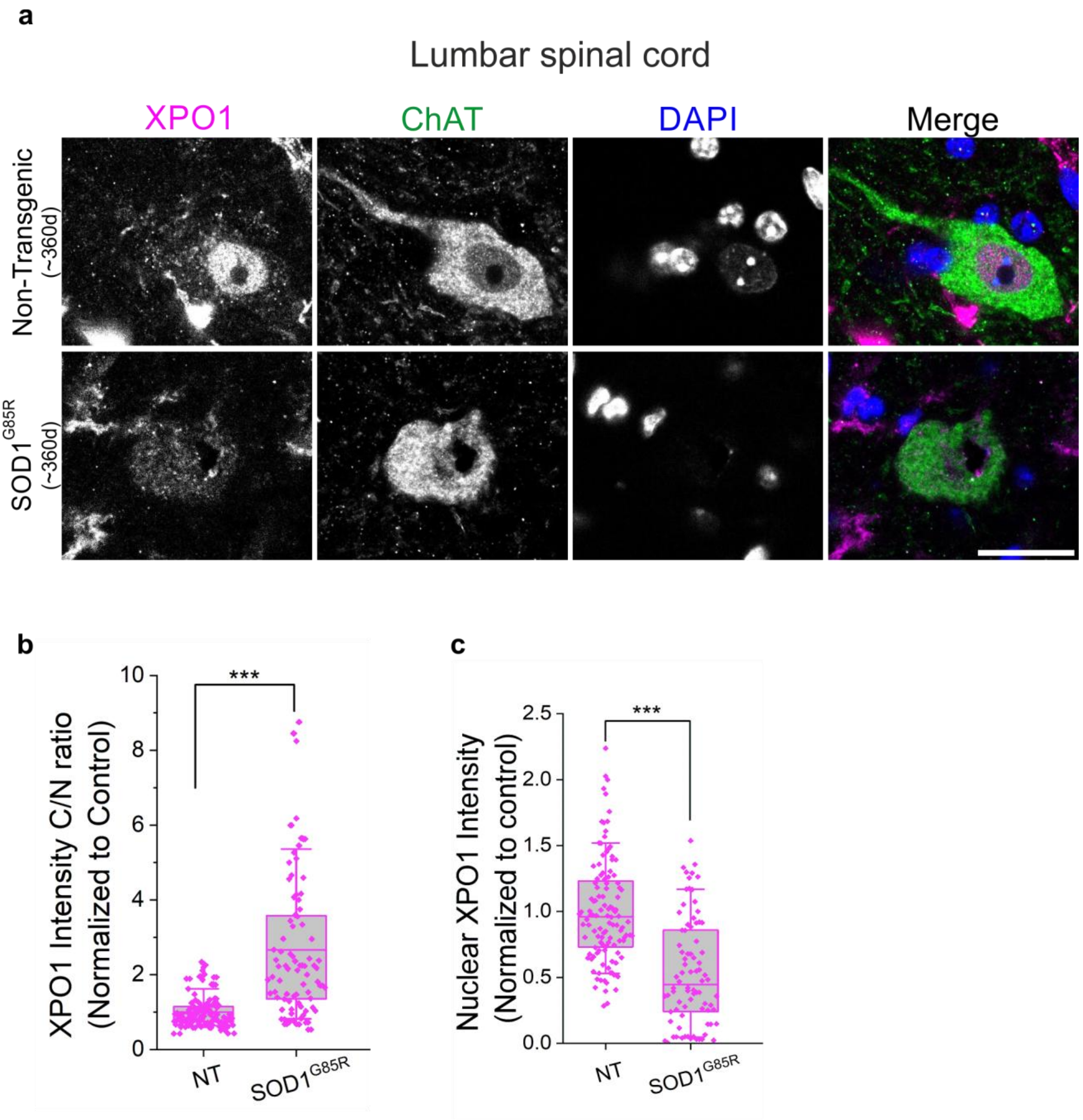

Supplementary Figure 2

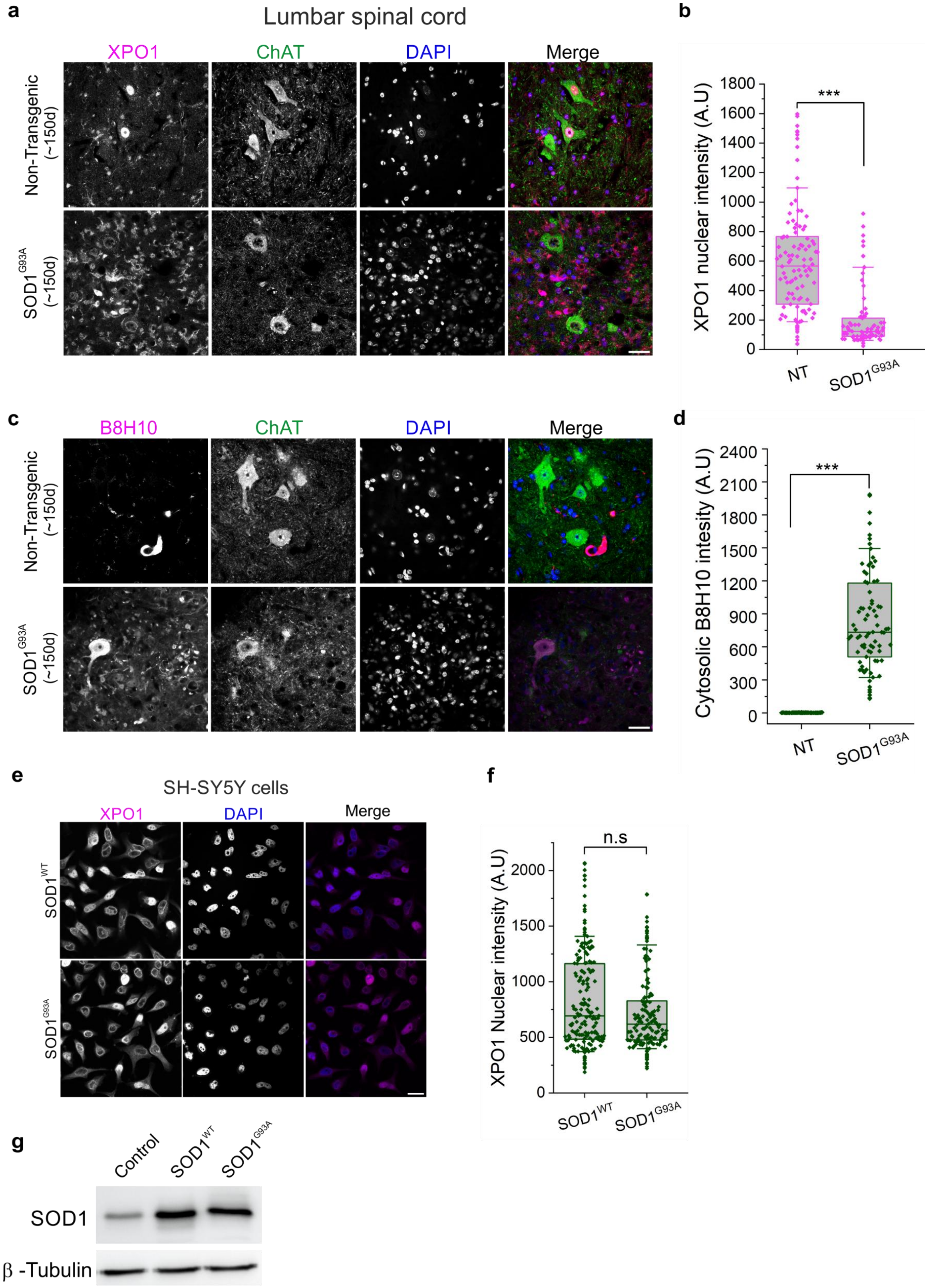

Supplementary Figure 3

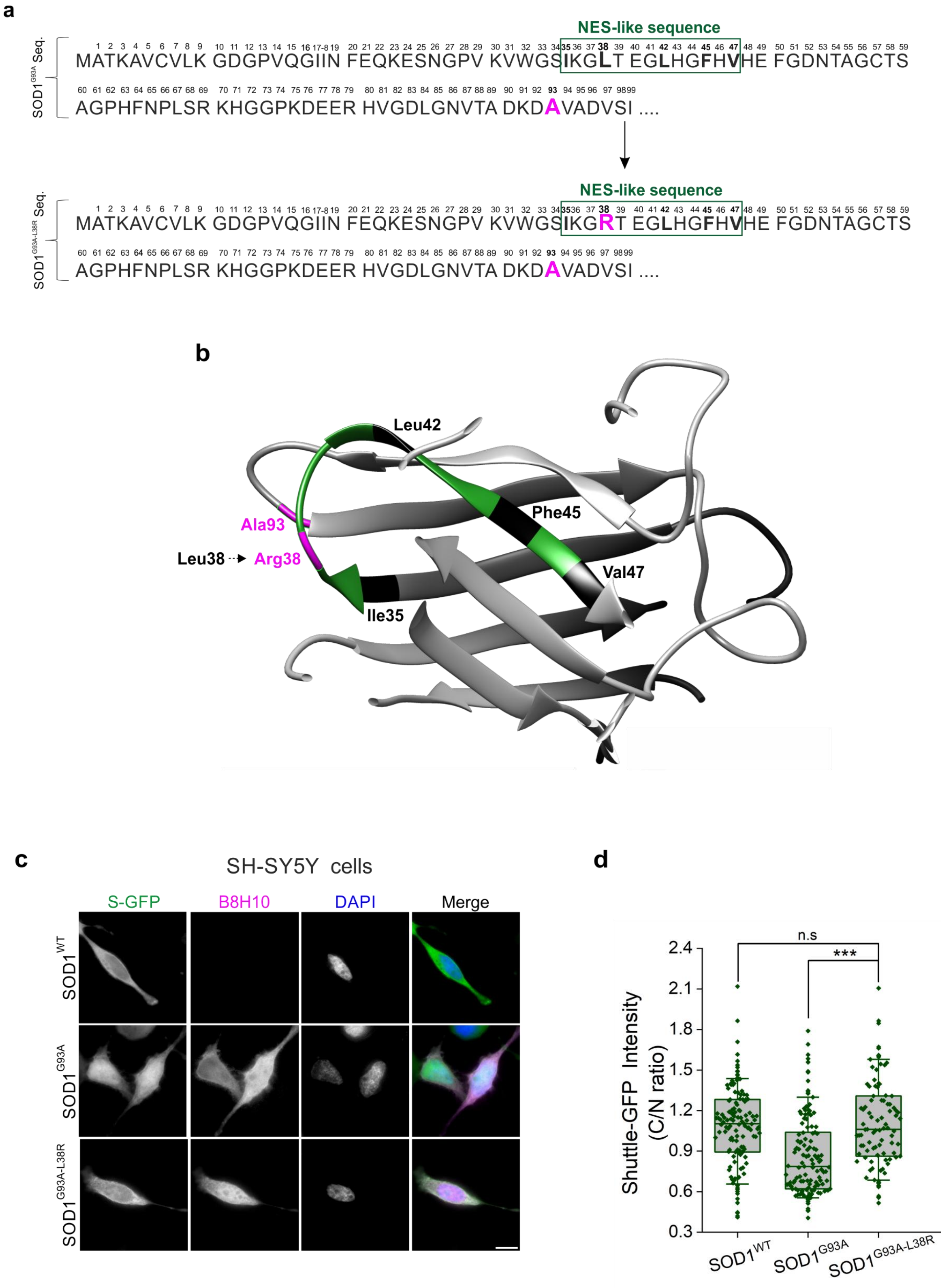

Supplementary Figure 4

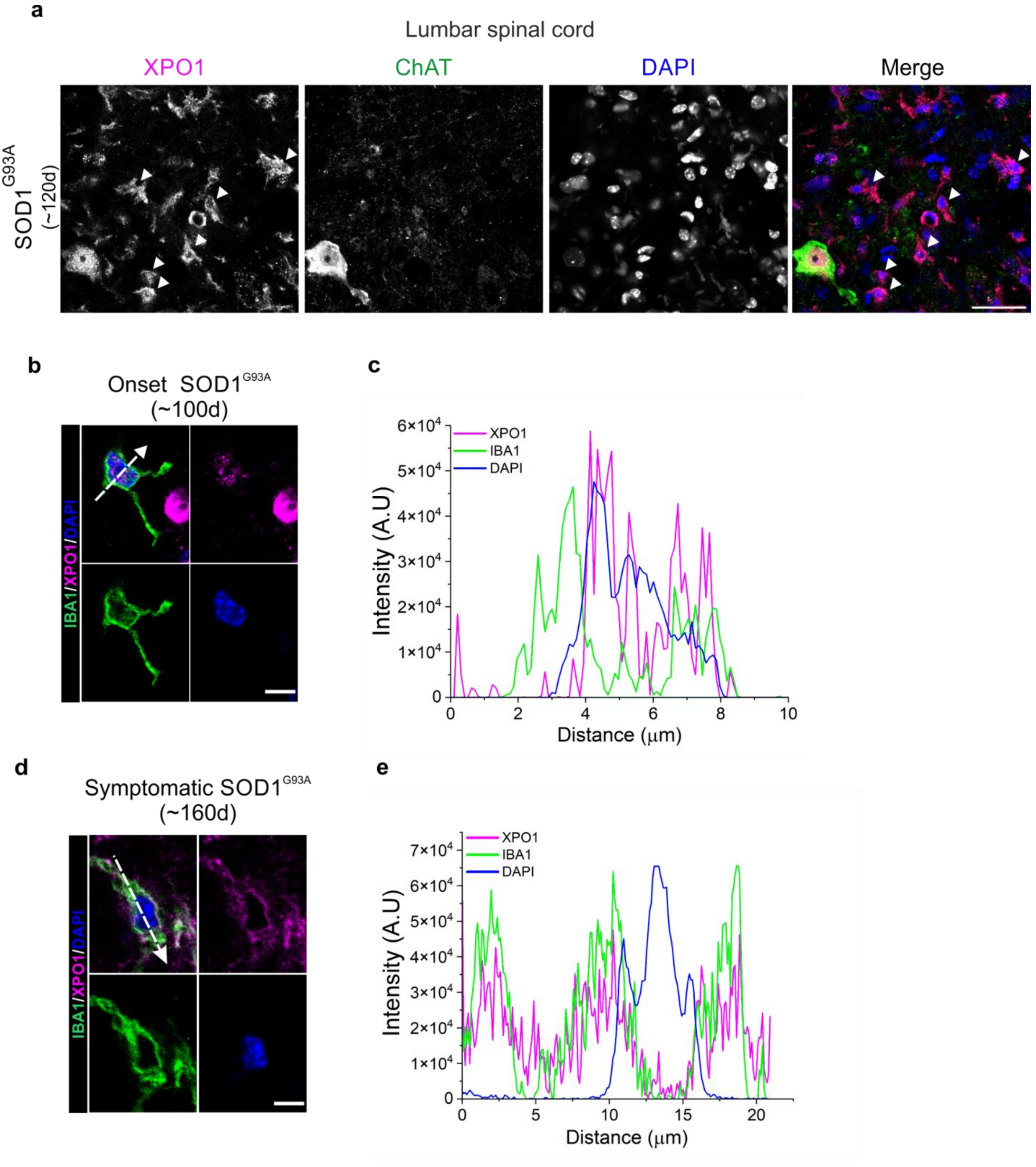

Supplementary Figure 5

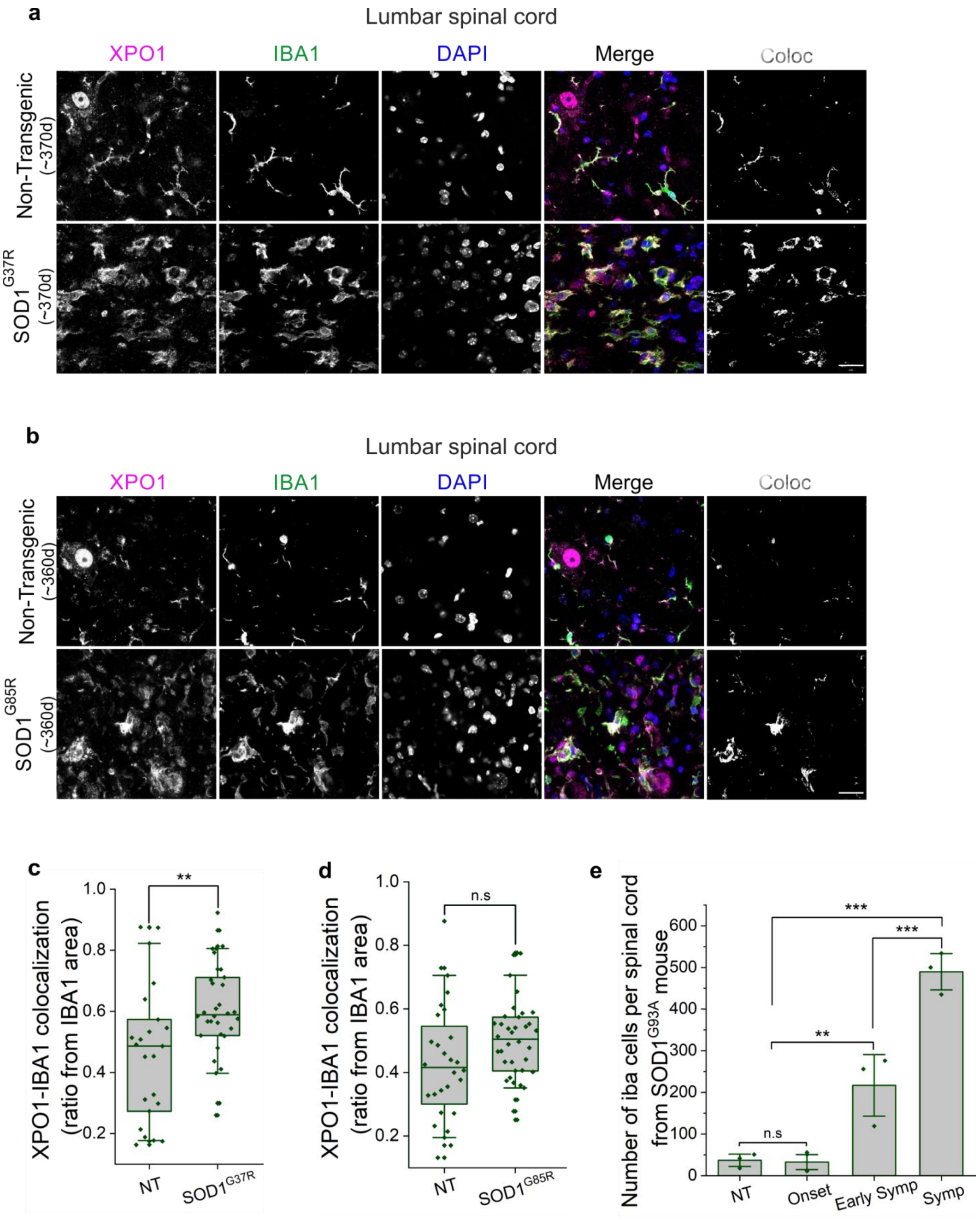

Supplementary Figure 6

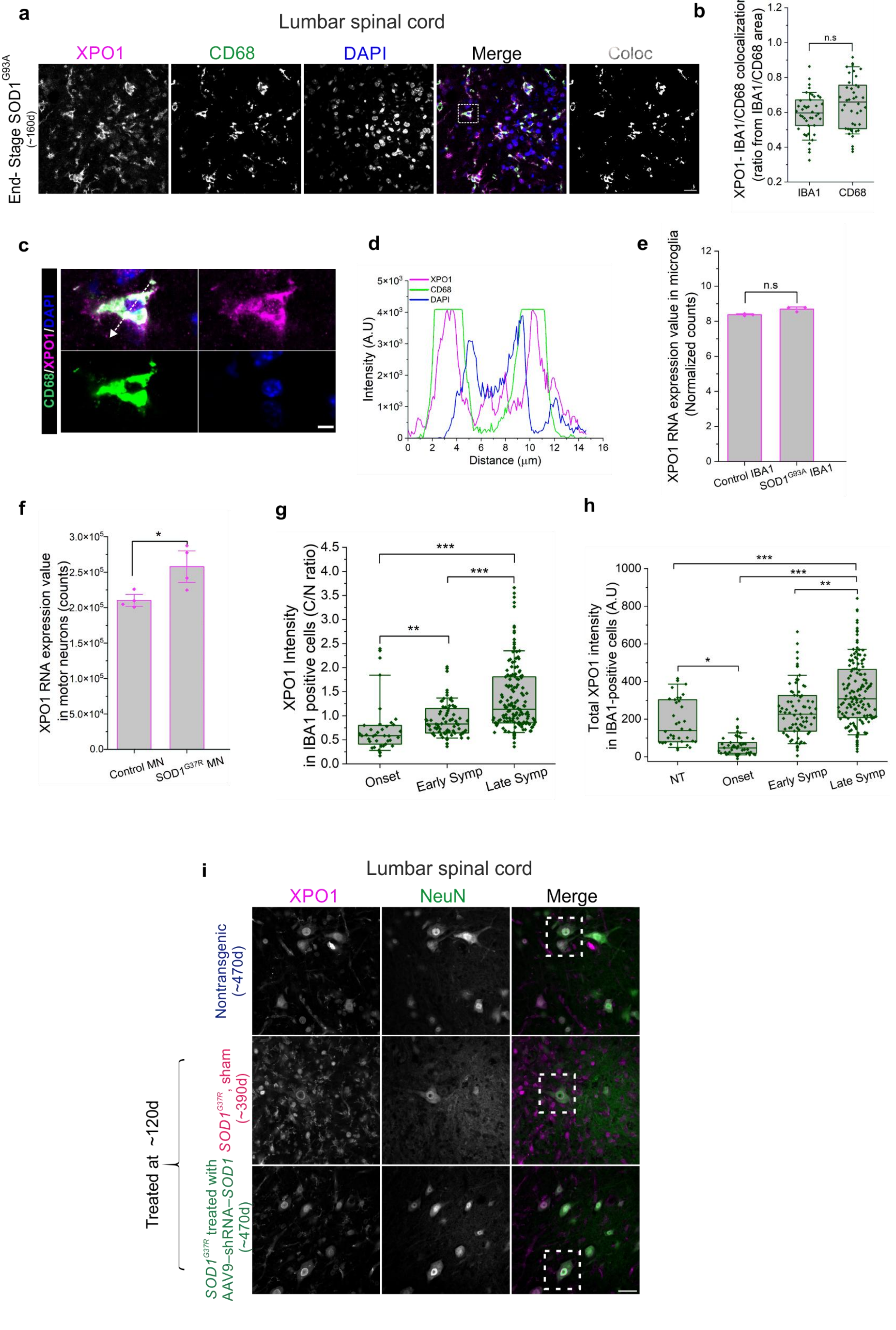

Supplementary Figure 7

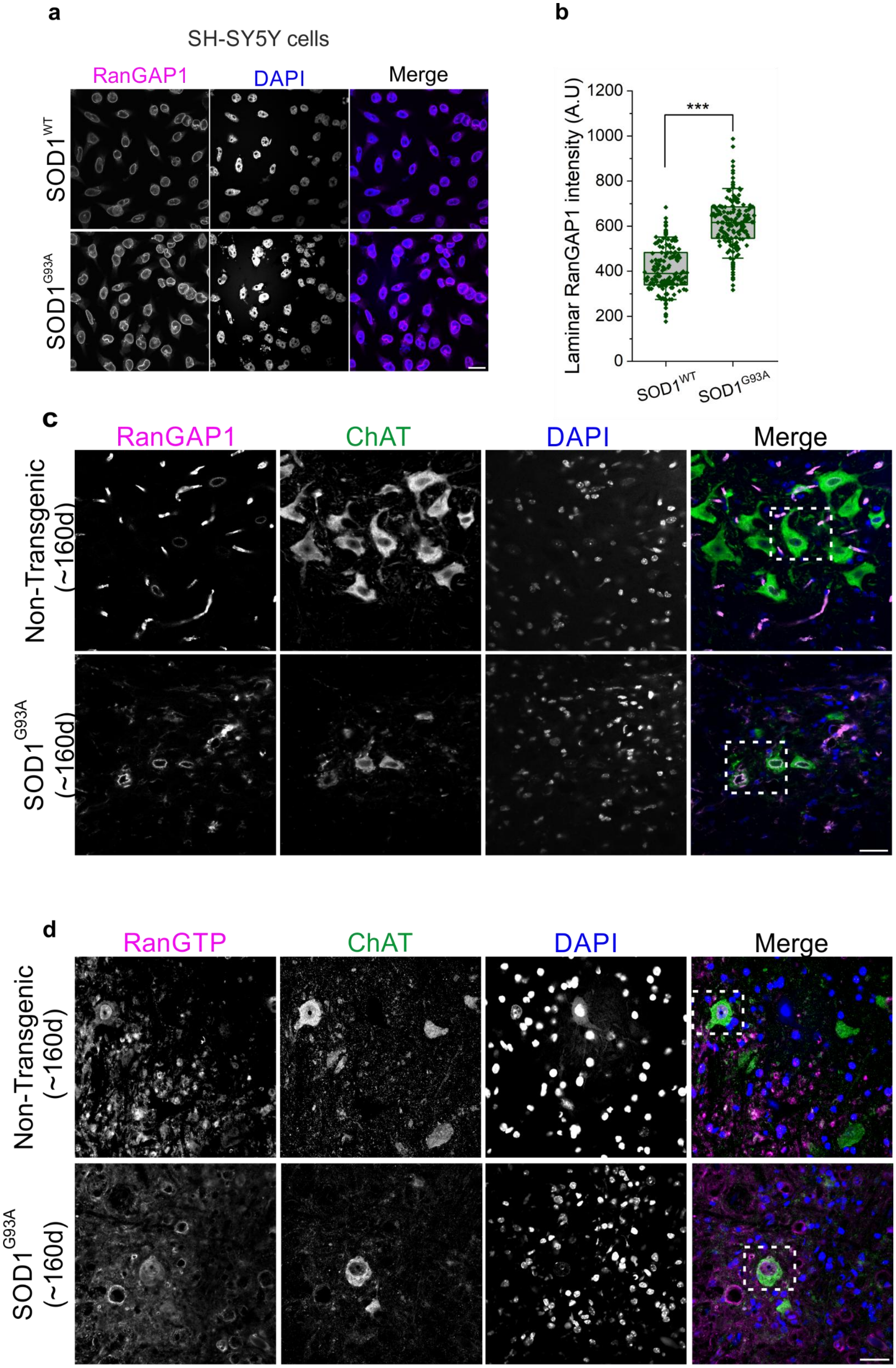

Supplementary Figure 8

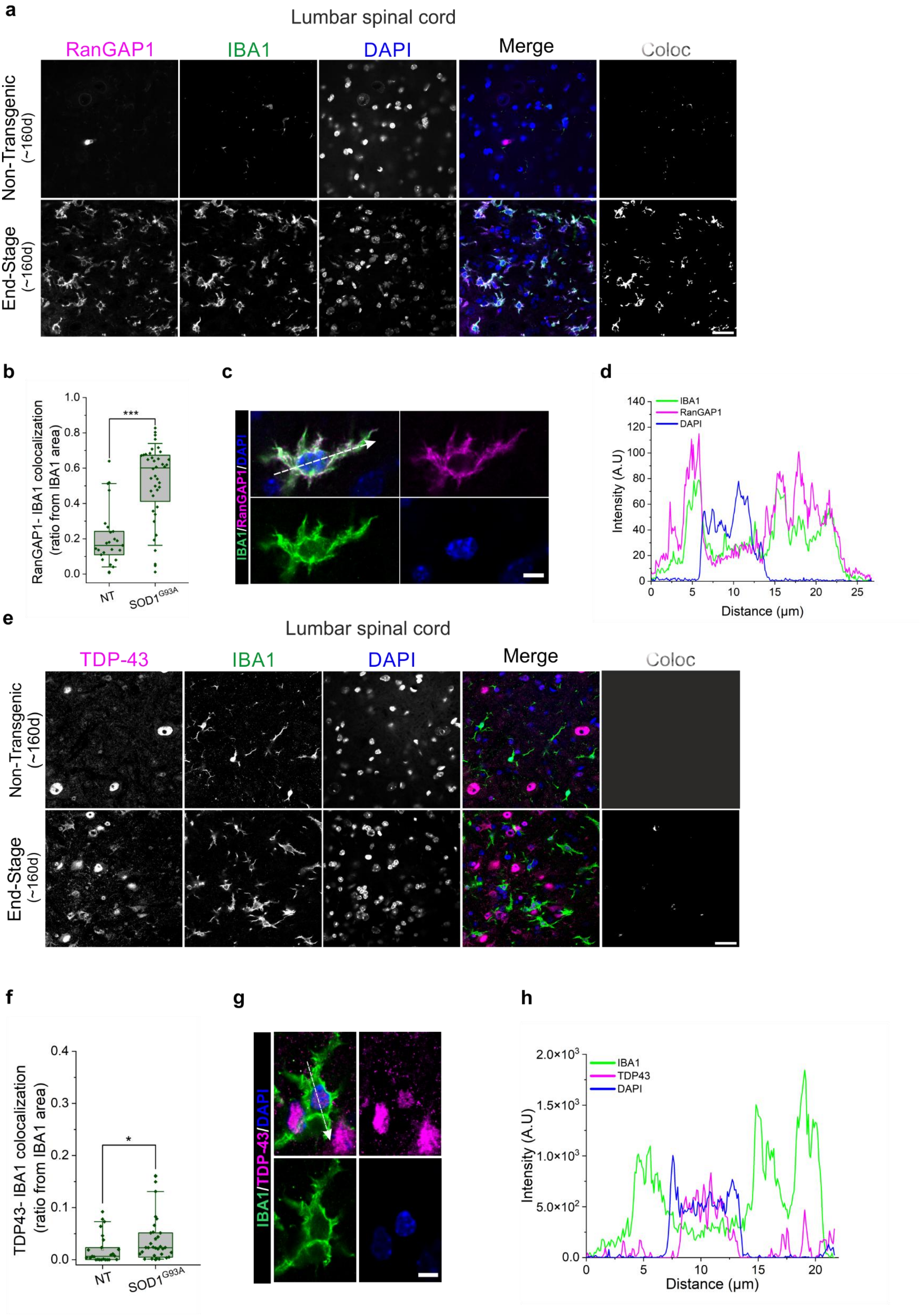

Supplementary Figure 9

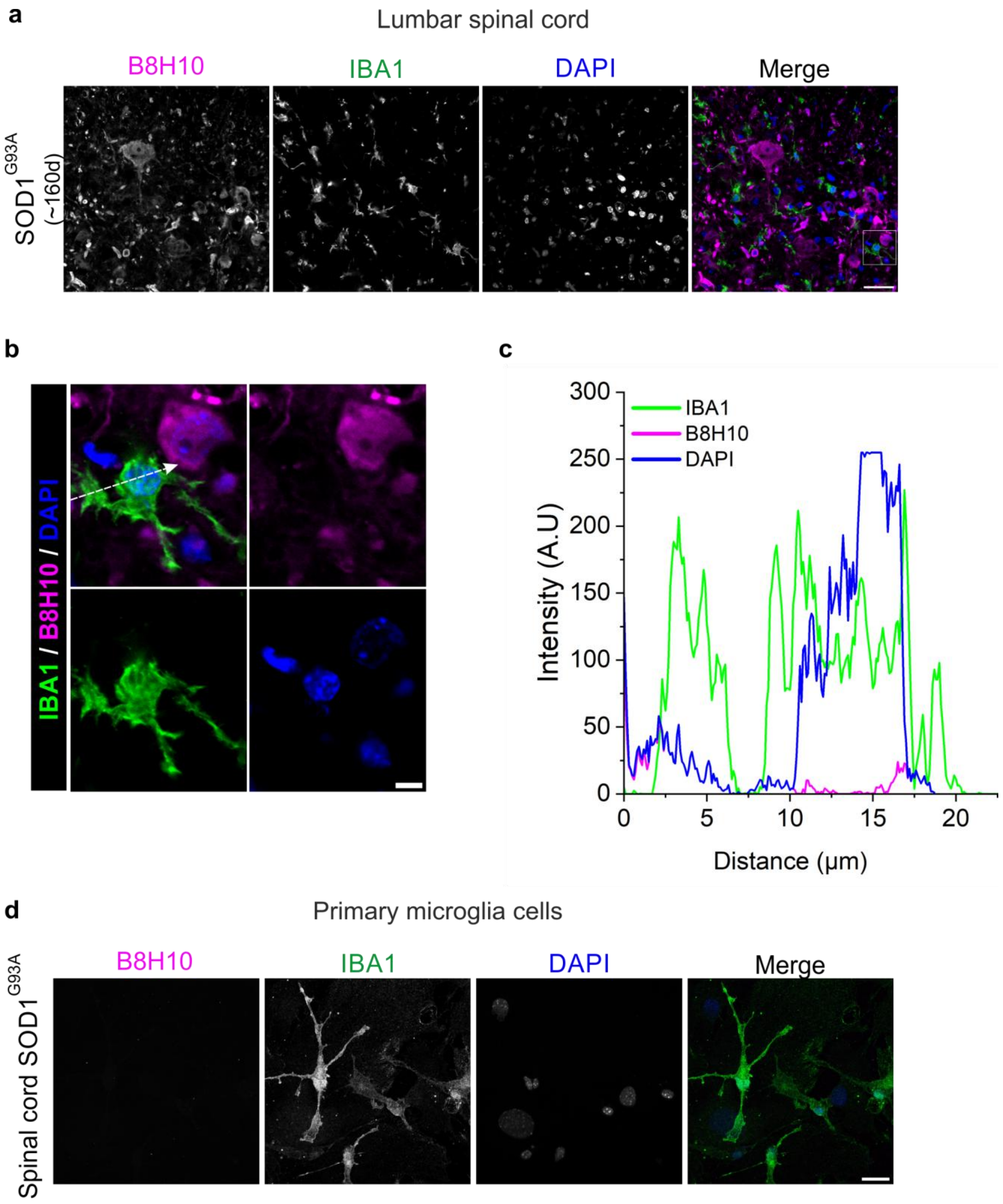

Supplementary Figure 10

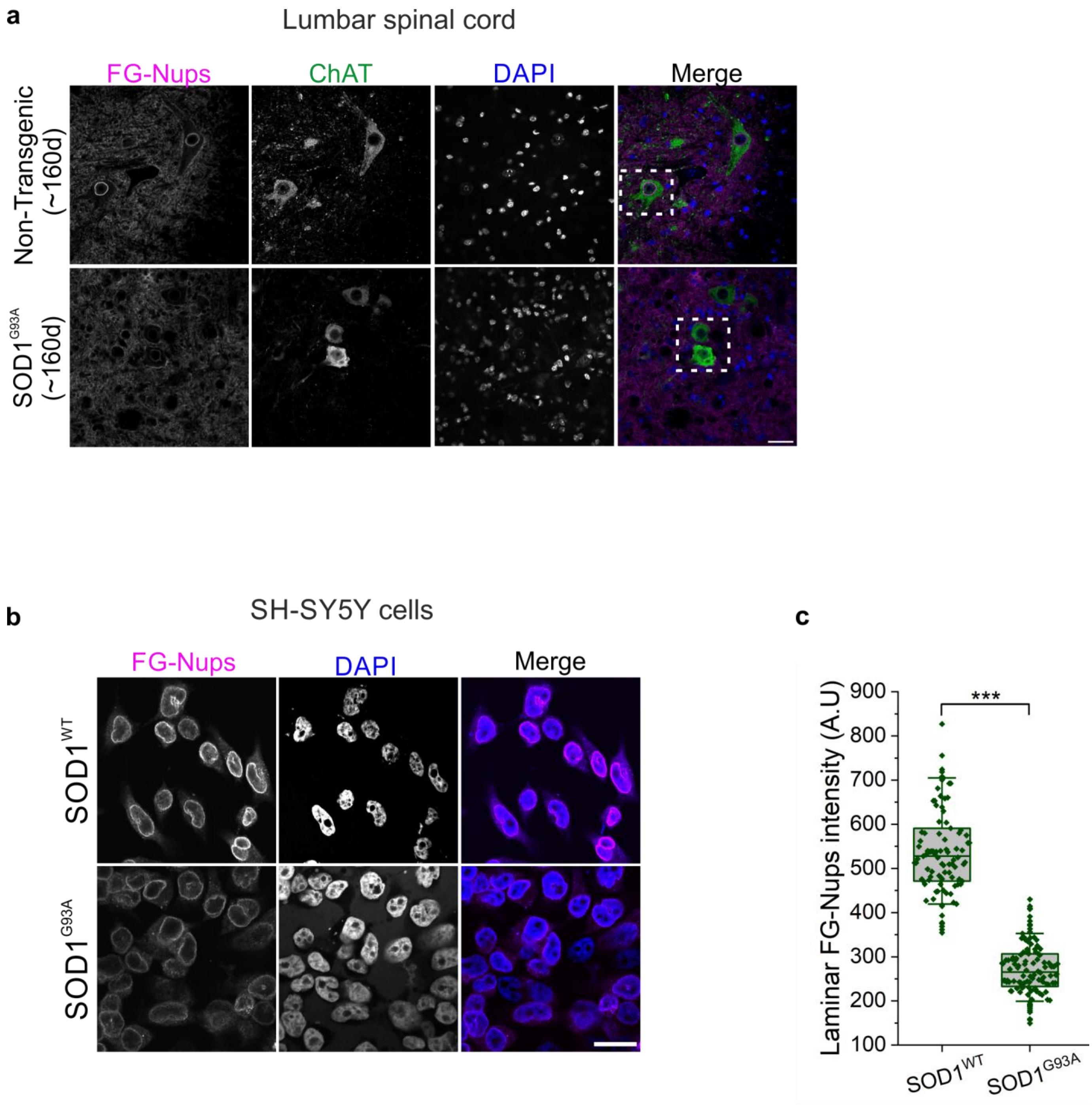

Supplementary Figure 11

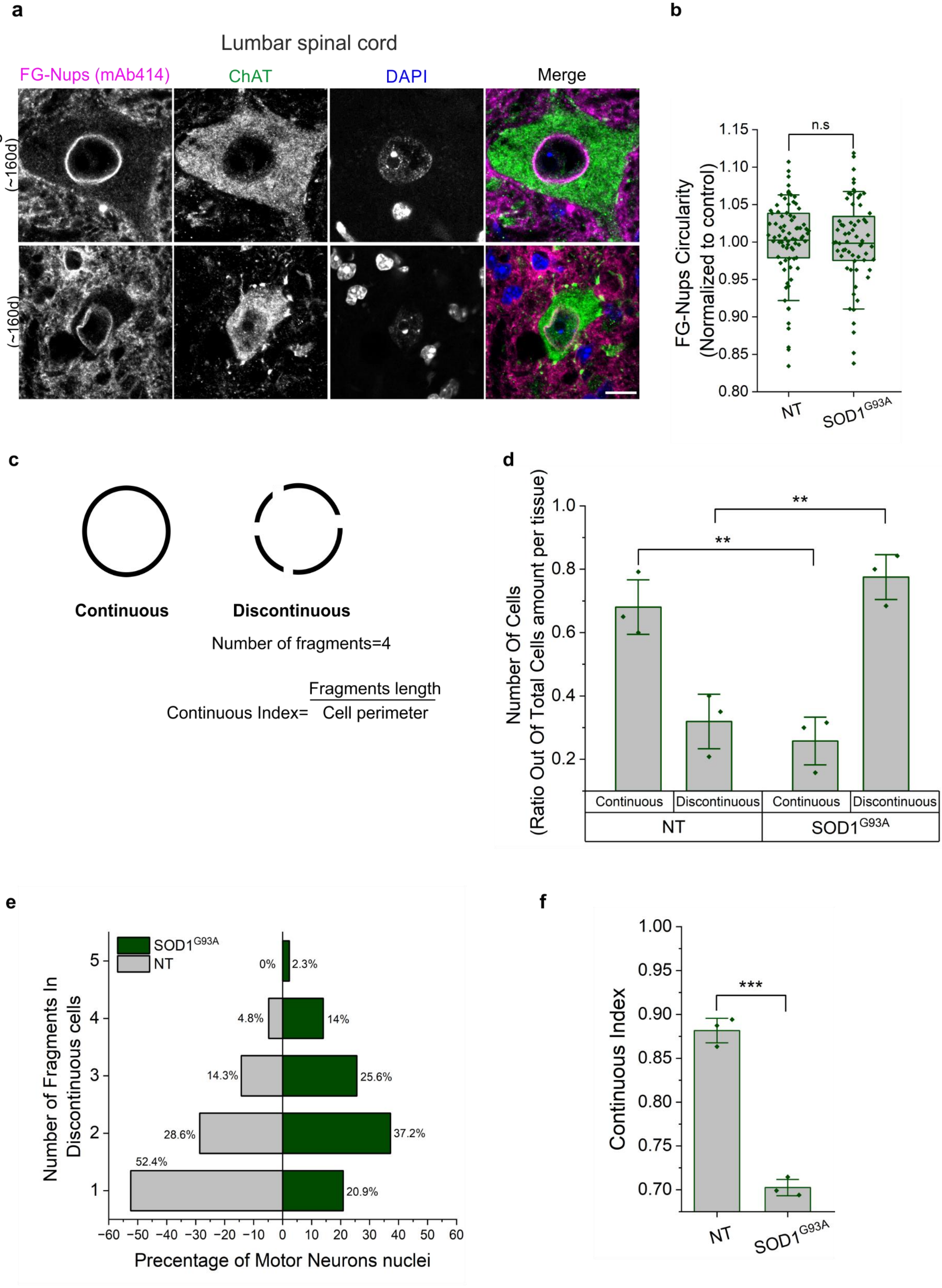

Supplementary Figure 12

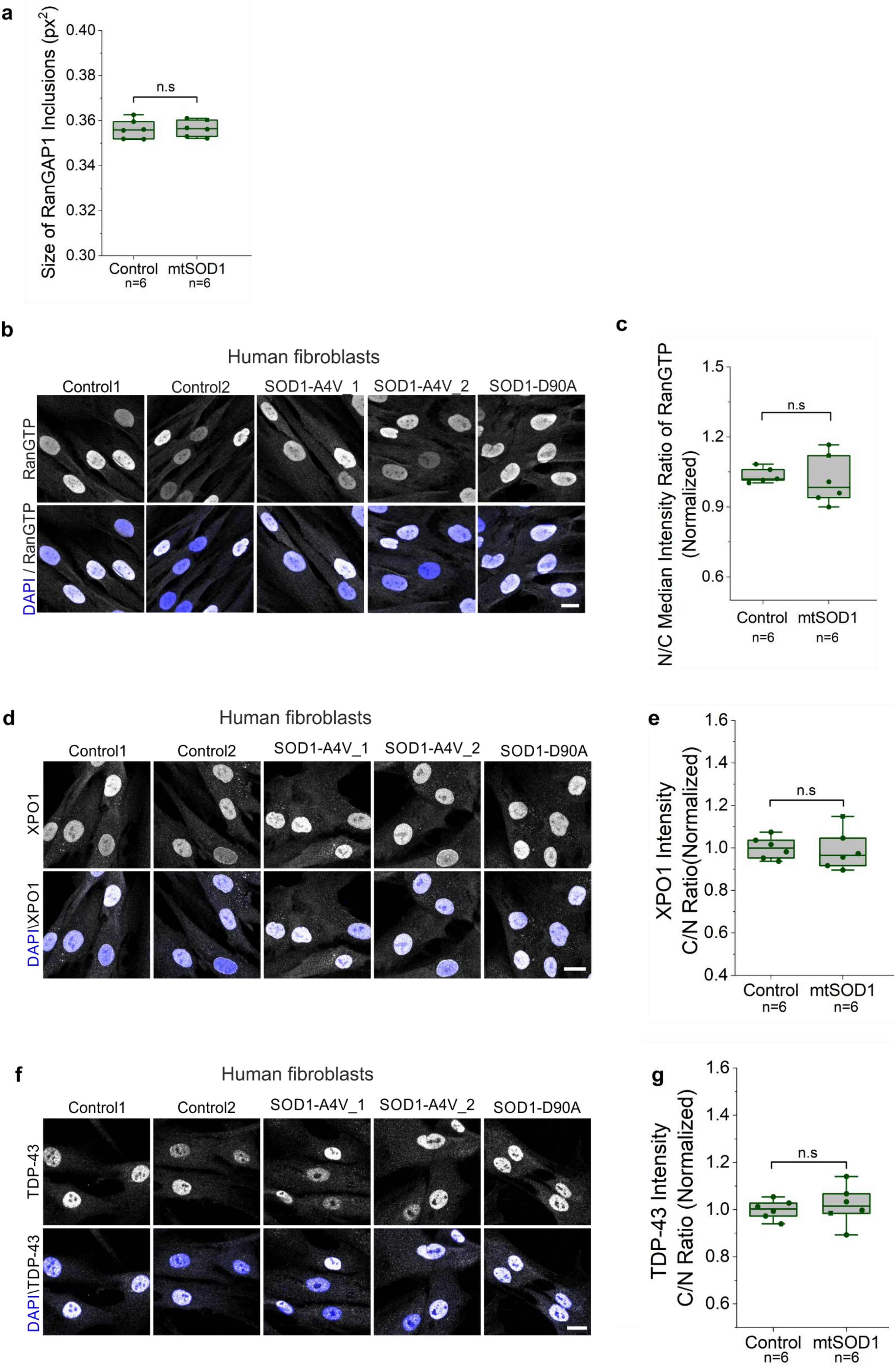

Supplementary Figure 13

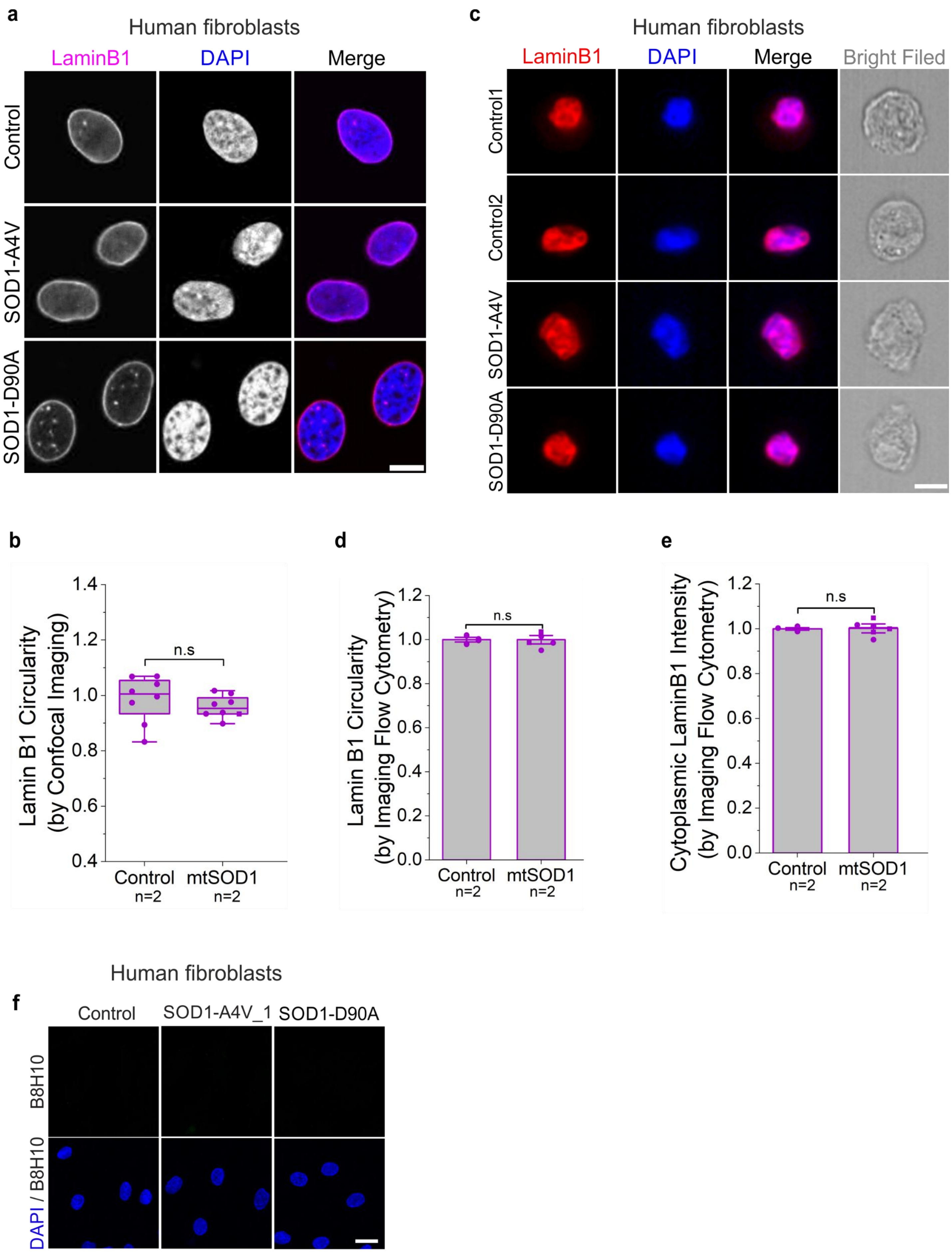

Supplementary Figure 14

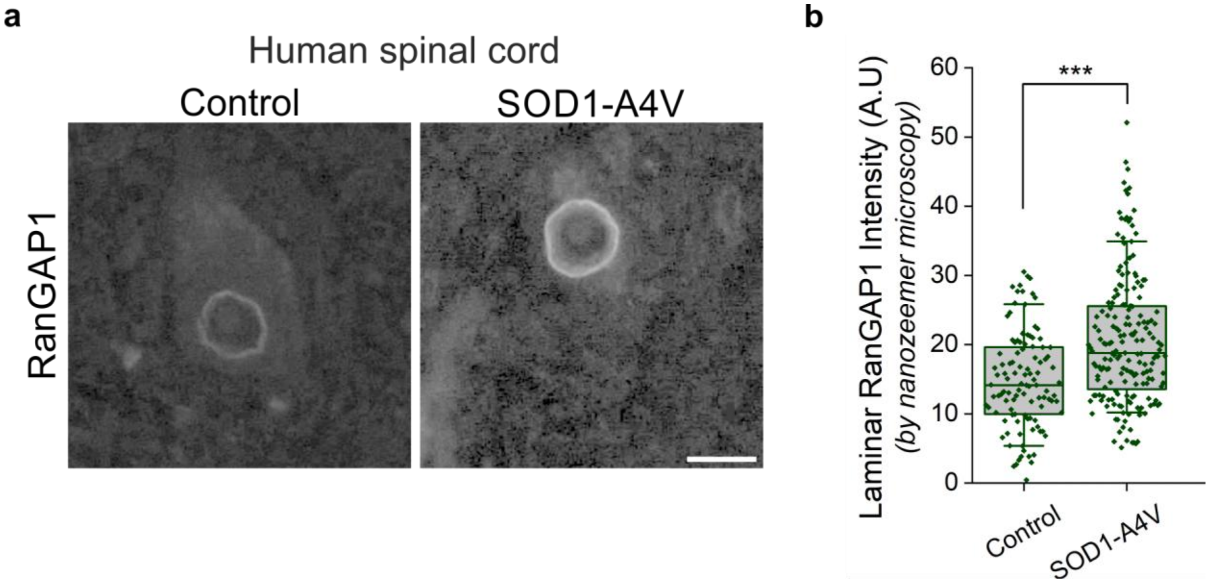

Supplementary Figure 15
