# Supplementary figures and images for: Impaired nucleocytoplasmic transport in SOD1-mediated ALS
Source: Mol Neurodegener. 2026 Feb 14;21:14. doi: 10.1186/s13024-026-00930-8 (PMC12922372; doi:10.1186/s13024-026-00930-8)

SOD1 →

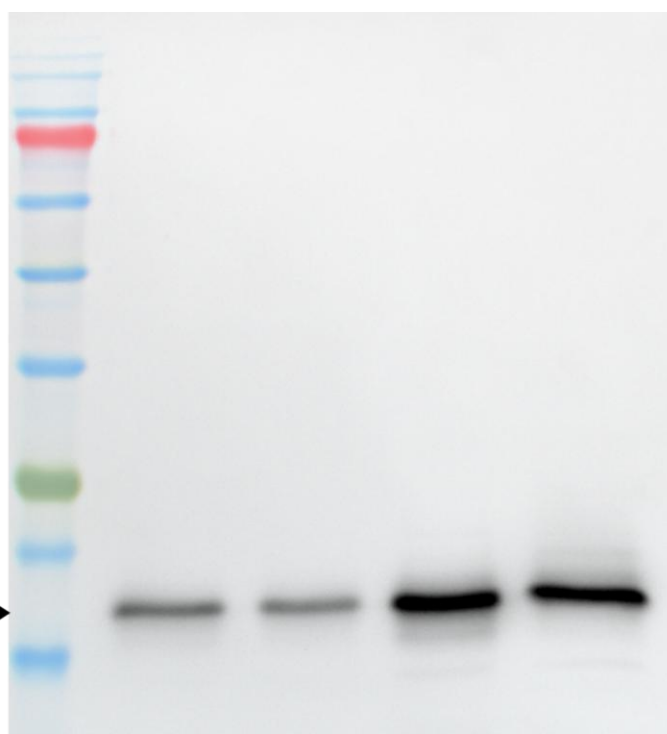

$\beta$ -Tubulin →

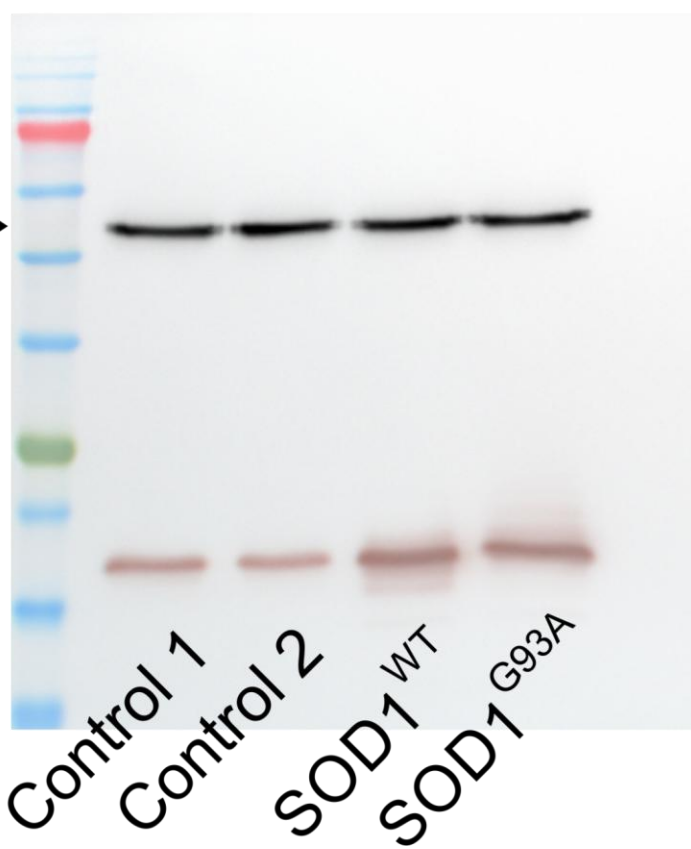

Supplement: Supplementary file 3 — Supplementary Material 3 [file 13024_2026_930_MOESM3_ESM.pdf]
